# Assessing the causal role of hypertension on left atrial and left ventricular structure and function: A two-sample Mendelian randomization study

**DOI:** 10.3389/fcvm.2022.1006380

**Published:** 2022-11-02

**Authors:** Yancui Sun, Ying Zhang, Nan Xu, Cheng Bi, Xiaojie Liu, Wei Song, Yinong Jiang

**Affiliations:** ^1^Department of Cardiology, First Affiliated Hospital of Dalian Medical University, Dalian, Liaoning, China; ^2^School of Basic Medical Sciences, Henan University, Kaifeng, Henan, China; ^3^Department of Cardiology, Tieling Central Hospital, Tieling, Liaoning, China; ^4^Department of Cardiology, The Liaoyu Hospital of Dalian, Dalian, Liaoning, China

**Keywords:** left atrial, left ventricular, Mendelian randomization, hypertension, genetics

## Abstract

**Aim:**

The aim of this study was to investigate whether hypertension may be causally linked to left atrial (LA) and left ventricular (LV) structure and function.

**Methods and results:**

We performed a two-Mendelian randomization (MR) analysis implementing the results from the FinnGen large-scale, genome-wide association study for hypertension (*N* = 218,754), and LV (*N* = 16,923) and LA studies (*N* = 35,648) by the UK Biobank to identify genetic instruments. The MR analysis was implemented using an inverse-variance weighted (IVW) approach. We identified a positive potential causal relationship between hypertension and indices for the LA maximum (LAmax with causal estimates of 0.126 [95% CI, (0.093 to 0.160)]); LA minimum (LAmin with causal estimates of 0.122 [95% CI, (0.089 to 0.156)]); LV function (causal estimates are LV end-diastolic volume (LVEDV), 0.078 [95% CI, (0.003 to 0.153)]; LV end-systolic volume (LVESV), 0.102 [95% CI, (0.030 to 0.173)]; LV mass (LVM), 0.171 [95% CI, (0.108 to 0.233)]; and LV mass to end-diastolic volume ratio (LVMVR at 0.098 [95% CI, (0.048 to 0.149)], respectively), which was directionally concordant with other robust MR methods. Other than this, we observed a significantly negative causal relationship between hypertension and the LA active emptying fraction (LAAEF), the LA passive emptying fraction (LAPEF), and the LA total emptying fraction (LATEF).

**Conclusion:**

Our genetic analyses demonstrated a potential causal relationship between hypertension and the left atrium and left ventricle’s structures and functions.

## Introduction

Hypertension is a prevalent cardiovascular disorder affecting millions of people worldwide. Long-term hypertension can lead to increased cardiac afterload, often causing myocardial morphological and structural changes, including left atrial (LA) dilatation and left ventricular (LV) hypertrophy. The enlargement volume and dysfunction of the left atrium are important compensatory behaviors for changes in critics of the LA average pressure or the increased preload; in addition, the increases in LA volume and dysfunction are parallel to the degree of LV dysfunction (1). There are many studies on the relationship between hypertension and LV/LA structure and function. Jung et al. (2) performed a large cohort study among 52,111 Korean participants, revealing a significant association between hypertension and the risk of LV remodeling. The Pressioni Monitorate E Loro Associazion (PAMELA) study reported a similar conclusion of progressive increase from normotensive to pre-hypertension and hypertension groups in the incidence of LV hypertrophy (3). In recent years, there have been several articles in cardiology literature on hypertension and LA size and function (4, 5). A genetic study about the LA, initiated by Ahlberg et al. (6), showed that systolic blood pressure had a significant effect on LA volume and function. Although the association between hypertension and LA/LV and hypertension has been widely studied. However, hypertension tends to coincide with other cardiovascular risk factors, such as obesity and smoking, thereby making it difficult to identify the independent effects that blood pressure has on the heart’s structure and function. Thus, it is necessary to disentangle the causal relationship between hypertension and the left heart system from the perspective of genetics.

As an emerging method used for causal inference in epidemiology, Mendelian randomization (MR) has achieved great success. Gregor Mendel proposed two laws of inheritance in 1866: Random assignment and free combination. These laws are summed up with the phrase, “Mendelian laws of inheritance.” Resulting in the name of MR. In modern literature, MR has become a popular method for assessing causal effects (7, 8). It uses genetic variation as an instrumental variable to determine the causal relationship between exposure and outcomes, an approach that can effectively avoid reverse causation or confoundment.

Our study involved a two-sample MR analysis to explore the causal associations between hypertension and LA and LV structure and function by using recent genome-wide association studies (GWASs) and corroborating previous findings.

## Methods

### Mendelian randomization design

**FIGURE 1:**
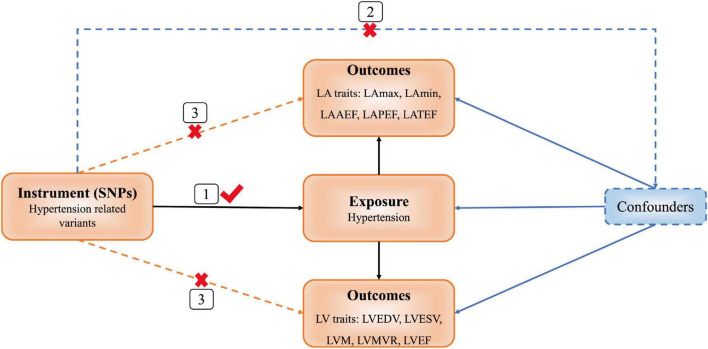
Diagram of Mendelian randomization (MR) framework. SNP, single nucleotide polymorphisms; LA, left atrial; LAmax, LA maximum volumes; LAmin, LA minimum volumes; LAAEF, LA active emptying fraction; LAPEF, LA passive emptying fraction; LATEF, LA total emptying fraction; LV, left ventricular; LVEDV, LV end-diastolic volume; LVESV, LV end-systolic volume; LVM, LV mass; LVMVR, LV mass to end-diastolic volume ratio; LVEF, LV ejection fraction.

We used a two-sample MR design supported by three hypotheses (9). First, instrumental variables [(single nucleotide polymorphisms (SNPs)] and exposure factor (hypertension) are strongly associated with each other. Second, instrumental variables and confounding factors are not correlated. Third, instrumental variables are associated with outcomes (LA/LV structure and function) only through hypertension, and not through other pathways (Figure 1).

### Data

We extracted the genetic associations of the instrumental variables with hypertension from the FinnGen study.^1^ This Finnish study was a nationwide GWAS meta-analysis of 13 cohorts and biobanks. The cohorts have been linked with longitudinal digital health record data from nationwide health registries. The data on hypertension included 55,917 case samples and 162,837 control samples.

We searched PubMed for GWAS involving LA/LV structure and function, and identified genetic variants for LA indices (6) and LV indices (10). We found cardiac phenotypes derived from the participants with cardiovascular magnetic resonance imaging (CMR) in the UK Biobank (11). The UK Biobank was a cohort study conducted between 2006 and 2010 that included over 500,000 men and women from the UK general population. The LV GWAS study included 16,923 European UK Biobank participants (48.5% were men with a mean age of 62.5 years) who had not had a myocardial infarction or heart failure (HF). There were 35,648 participants in the LA GWAS study; exclusion criteria included previous myocardial infarction, a diagnosis of HF or cardiomyopathy, and a body mass index (BMI) <16 or >40 kg/m^2^.

The following traits of LA/LV structure and function were derived in the following manner: indexed LA maximum (LAmax) and LA minimum (LAmin) volumes, LA active emptying fraction (LAAEF), LA passive emptying fraction (LAPEF), LA total emptying fraction (LATEF), LV end-diastolic volume (LVEDV), LV end-systolic volume (LVESV), LV ejection fraction (LVEF), LV mass (LVM), and LV mass to end-diastolic volume ratio (LVMVR). The LVM was determined at the end-diastolic phase. The LVMVR was calculated by dividing LVM by LVEDV and assessing the LV concentricity (12).

### Statistical analysis

To verify that the SNPs selected in this study met the assumptions, we examined the genetic association between hypertension and measured linkage disequilibrium (LD) among all the SNPs, and selected independent genetic variants for hypertension. Plink version 1.9 was implemented with the genome-wide significance level (*P* < 5 × 10^–8^), *r*^2^ < 0.001 and kb = 500 in order to select 70 independent SNPs with hypertension. Then, we excluded SNPs that were related to other traits (including BMI, coronary heart disease and diabetes mellitus), GWAS^2^ and those that had a significant association with cardiac outcomes [<0.05/n. SNP (hypertension)] before MR analysis.

Next, we performed the MR analysis using the MR package. The inverse-variance weighted (IVW) methods (13) were performed to evaluate the causal association between hypertension and LA/LV, with other MR methods such as the weighted median method, the MR-Egger regression (14) and the MR pleiotropy residual sum and outlier method (MR-PRESSO) (15) being carried out as complementary and sensitivity analyses. The MR-Egger intercept was used to identify any potential directional pleiotropy. The MR-PRESSO method was used to identify outlying SNPs that were potentially horizontally pleiotropic, and to check whether their exclusion changed the causal estimate. If the outliers were detected, they would be removed, and we would reassess the MR causal estimation. The MR-PRESSO-corrected results are reported in the main results as well, as they adopted the IVW method. We also calculated the F-statistic to test instrument strength (*F* > 10 indicated sufficient strength) (16). Cochran’s *Q*-test was used to assess the heterogeneity (17).

All of the analyses were performed using R software version 4.1.2 and Plink version 1.9. These MR analyses were conducted with the MR package version 0.4.3 (18) and MR-PRESSO (15).

## Results

### Mendelian randomization and sensitivity analysis of hypertension and left atrial indices

Among the 70 SNPs associated with hypertension, we chose 53, 54, 55, 55, and 55 correlated hypertension instrumental variables to evaluate their associations with LAmax, LAmin, LAAEF, LAPEF, and LATEF, respectively. This information is summarized in Supplementary Tables 1–5. The F-statistics for these SNPs were all >31, suggesting that weak instrument bias was potentially null in our analysis. The Cochran’s *Q*-test showed that there was no evidence of instrumental heterogeneity for hypertension (*P* > 0.05), as shown in Table 1. Therefore, we employed the fixed-effects IVW method for hypertension when estimating the causal effects on LAmax, LAmin, LAAEF, LAPEF, and LATEF.

**TABLE 1:** Estimated effect size of hypertension and LA with different MR methods.

	SNPs	Method	Beta (Se)	*P*	Heterogeneity test P
LAmax	53	IVW (fixed effect)	0.126 (0.017)	1.59E-13	0.109
		IVW (random effect)	0.126 (0.019)	3.90E-11	
		Weighted median	0.141 (0.026)	6.01E-08	
		MR-Egger	0.193 (0.059)	2.00E-03	
		MR-Egger intercept	–0.006 (0.005)	0.242	
	53	MR-PRESSO	0.126 (0.017)	1.59E-13	
LAmin	54	IVW (fixed effect)	0.122 (0.017)	6.24E-13	0.130
		IVW (random effect)	0.122 (0.019)	7.38E-11	
		Weighted median	0.123 (0.026)	1.56E-06	
		MR-Egger	0.114 (0.059)	0.058	
		MR-Egger intercept	0.001 (0.005)	0.888	
	54	MR-PRESSO	0.122 (0.017)	6.24E-13	
LAAEF	55	IVW (fixed effect)	–0.044 (0.017)	9.10E-03	0.072
		IVW (random effect)	–0.044 (0.019)	2.19E-02	
		Weighted median	–0.050 (0.026)	5.15E-02	
		MR-Egger	0.037 (0.060)	0.536	
		MR-Egger intercept	–0.007 (0.005)	0.157	
	55	MR-PRESSO	–0.044 (0.017)	9.10E-03	
LAPEF	55	IVW (fixed effect)	–0.105 (0.017)	5.15E-10	0.098
		IVW (random effect)	–0.105 (0.019)	2.91E-08	
		Weighted median	–0.134 (0.025)	8.80E-08	
		MR-Egger	–0.098 (0.060)	0.107	
		MR-Egger intercept	–0.001 (0.005)	0.906	
	55	MR-PRESSO	–0.105 (0.017)	5.15E-10	
LATEF	55	IVW (fixed effect)	–0.072 (0.017)	1.91E-05	0.068
		IVW (random effect)	–0.072 (0.019)	1.78E-4	
		Weighted median	–0.082 (0.026)	0.001	
		MR-Egger	0.018 (0.060)	0.763	
		MR-Egger intercept	–0.007 (0.005)	0.116	
	55	MR-PRESSO	–0.072 (0.017)	1.91E-05	

MR, Mendelian randomization; IVW, the inverse-variance weighted method; MR-PRESSO, MR Pleiotropy Residual Sum and Outlier; LA, left atrial; LAmax, LA maximum volumes; LAmin, LA minimum volumes; LAAEF, LA active emptying fraction; LAPEF, LA passive emptying fraction; LATEF, LA total emptying fraction.

**FIGURE 2:**
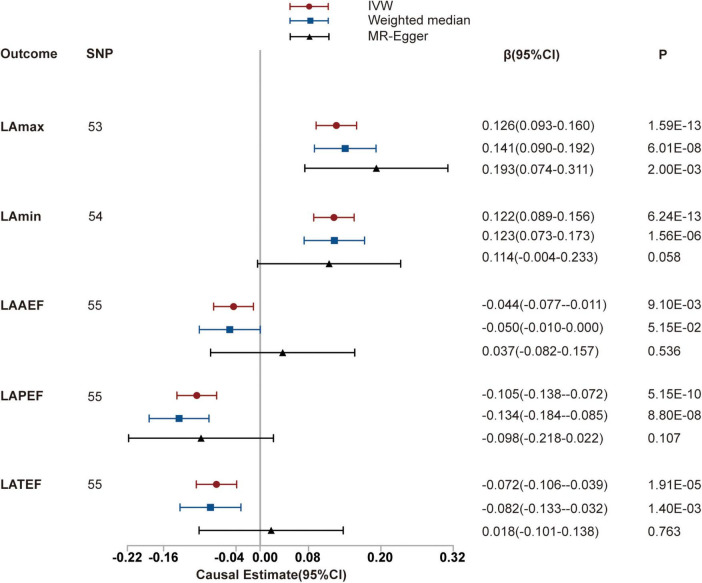
Mendelian randomization (MR) analyses testing the effect of hypertension on left atrial index. Results of three MR methods [inverse-variance weighted (IVW), MR-Egger and weighted median] are presented as causal estimates with 95% CIs.

We identified positive potential causal relationships between hypertension and LAmax and LAmin using causal estimates of β: (0.126 [95% CI, (0.093 to 0.160)] and 0.122 [95% CI, (0.089 to 0.156)], respectively). Otherwise, negative relationships were found between hypertension and LAAEF, LAPEF, and LATEF, with causal estimates of −0.044 [95% CI, (−0.077 to 0.011)], −0.105 [95% CI, (−0.138 to −0.072)] and −0.072 [95% CI, (−0.106 to −0.039)], respectively. The results of the sensitivity analyses (including weighted median and MR-PRESSO) were consistent. The MR-Egger intercept analysis showed no potential directional pleiotropy (all *P* > 0.05; Figure 2 and Table 1). The scatter plots and funnel plots are presented in Supplementary Figures 1, 2.

### Mendelian randomization and sensitivity analysis of hypertension and left ventricular indices

**FIGURE 3:**
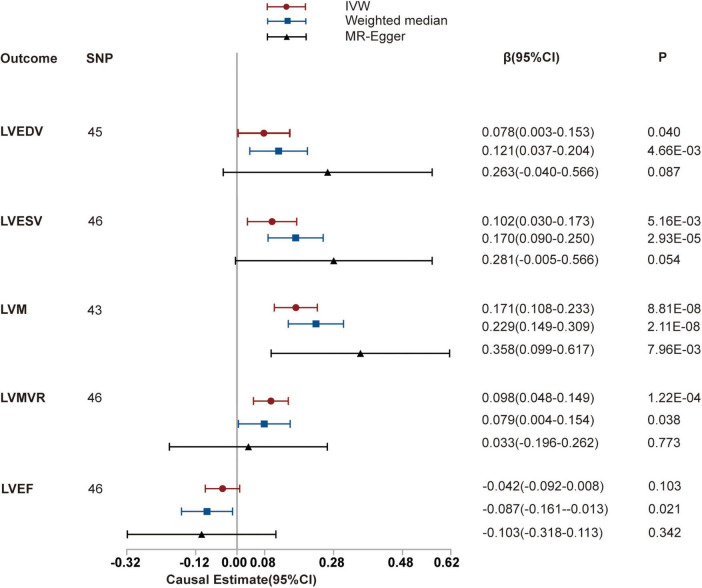
Mendelian randomization (MR) analyses testing the effect of hypertension on left ventricular index. Results of three MR methods [inverse-variance weighted (IVW), MR-Egger and weighted median] are presented as causal estimates with 95% CIs.

We then investigated the causal relationships between hypertension and LVEDV, LVESV, LVM, LVMVR, and LVEF using 45, 46, 43, 46, and 46 uncorrelated SNPs as instrumental variables for analysis, respectively. These details are shown in Supplementary Tables 6–10. Thereafter, we used the random-effects IVW method to estimate the causal effects resulting from the heterogeneity observed for LVEDV, LVESV, and LVM of instruments (the *P*-values of the Cochran’s *Q*-test were < 0.05) and the fixed-effects IVW method to estimate the causal effects between hypertension and LVMVR and LVEF (the *P*-values of the Cochran’s *Q*-test were >0.05). As shown in Table 2 and Figure 3, hypertension is positively associated with LVEDV (causal estimates: 0.078 [95% CI, (0.003 to 0.153)]), LVESV (causal estimates: 0.102 [95% CI, (0.030 to 0.173)]), LVM (causal estimates: 0.171 [95% CI, (0.108 to 0.233)]) and LVMVR (causal estimates: 0.098 [95% CI, (0.048 to 0.149)]). However, no association was observed between hypertension and LVEF. Supplementary Figures 3, 4 show scatter plots and funnel plots.

**TABLE 2:** Effect size of hypertension and LV with different MR methods.

	SNPs	Method	Beta (Se)	*P*	Heterogeneity test P
LVEDV	45	IVW (fixed effect)	0.078 (0.026)	2.83E-03	2.28E-05
		IVW (random effect)	0.078 (0.038)	4.01E-02	
		Weighted median	0.121 (0.043)	4.66E-03	
		MR-Egger	0.263 (0.150)	0.087	
		MR-Egger intercept	–0.014 (0.011)	0.211	
	43	MR-PRESSO	0.088 (0.037)	1.89E-02	
LVESV	46	IVW (fixed effect)	0.102 (0.026)	6.81E-05	5.52E-05
		IVW (random effect)	0.102 (0.036)	5.16E-03	
		Weighted median	0.170 (0.041)	2.93E-05	
		MR-Egger	0.281 (0.142)	5.37E-02	
		MR-Egger intercept	–0.014 (0.011)	0.198	
	43	MR-PRESSO	0.138 (0.032)	1.50E-05	
LVM	43	IVW (fixed effect)	0.171 (0.027)	1.80E-10	3.75E-02
		IVW (random effect)	0.171 (0.032)	8.81E-08	
		Weighted median	0.229 (0.041)	2.11E-08	
		MR-Egger	0.358 (0.128)	0.008	
		MR-Egger intercept	–0.014 (0.010)	0.140	
	42	MR-PRESSO	0.181 (0.027)	2.02E-11	
LVMVR	46	IVW (fixed effect)	0.098 (0.026)	1.22E-4	0.110
		IVW (random effect)	0.098 (0.029)	6.35E-4	
		Weighted median	0.079 (0.038)	0.038	
		MR-Egger	0.033 (0.114)	0.773	
		MR-Egger intercept	0.005 (0.009)	0.556	
	46	MR-PRESSO	0.098 (0.026)	1.22E-4	
LVEF	46	IVW (fixed effect)	–0.042 (0.026)	0.103	0.283
		IVW (random effect)	–0.042 (0.027)	0.122	
		Weighted median	–0.087 (0.038)	2.08E-02	
		MR-Egger	–0.103 (0.008)	0.342	
		MR-Egger intercept	0.005 (0.008)	0.559	
	46	MR-PRESSO	–0.042 (0.026)	0.103	

MR, Mendelian randomization; IVW, the inverse-variance weighted method; MR-PRESSO, MR pleiotropy residual sum and outlier; LV, left ventricular; LVEDV, LV end-diastolic volume; LVESV, LV end-systolic volume; LVM, LV mass; LVMVR, LV mass to end-diastolic volume ratio; LVEF, LV ejection fraction.

We subsequently performed sensitivity analyses to validate the causal association previously observed. The results of the weighted median analysis were consistent with the IVW methods, and the MR-Egger intercept analysis showed that no LV indices had directional pleiotropy. However, the MR-PRESSO analysis showed that 2, 3, and 1 instrumental variables of hypertension with LVEDV, LVESV, and LVM, respectively, seemed to be potential outliers. Exclusion of outliers did not substantially alter our estimation of the causal effect (causal estimates: LVEDV, 0.088 [95% CI, (0.014 to 0.161)]; LVESV, 0.138 [95% CI, (0.075 to 0.200)]; and LVM, 0.181 [95% CI, (0.128 to 0.234)]).

## Discussion

Our study was a two-sample MR analysis about hypertension and its relationship to LA/LV structure and function. The study’s main findings were the observed associations between hypertension and increased LA volume (including LAmax and LAmin), LV structure (including LVEDV and LVESV) and LV function (including LVM and LVMVR), and decreased LA function (including LAAEF, LAPEF and LATEF). This provided evidence of a causal relationship between hypertension and the left heart system.

As the fourth greatest risk factor for deaths worldwide, hypertension has been a significant predictor of cardiovascular events (19, 20). Cardiac-targeted organ damage in patients with established hypertension presents mainly as LA enlargement and LV hypertrophy (21). As an active dynamic apparatus, the left atrium modulates LV fill volume and regulates the integration of cardiac performance. Studies in the general population have found that LA enlargement occurs in 22–27% of people with hypertension (22). Despite recent interest in the relationship between LA enlargement and hypertension, many studies have only evaluated the maximum LA volumes (23, 24). With the emergence of atrial cardiomyopathy, attention has focused on LA function (25). The left atrium has three main phasic functions in the sinus rhythm: a reservoir function (collecting and storing blood in systole), a conduit function (passively moving blood to the left ventricle in the early diastolic phase) and a booster pump function (actively pumping blood to the left ventricle in the late diastolic phase). Our MR analysis confirms a non-random overlap of genetic associations between hypertension and LA volume and function. LA volume levels, both LAmax and LAmin, were elevated in patients with hypertension. The LA function, including LAAEF, LAPEF, and LATEF, was decreased. These findings are in line with earlier studies showing a significant association between hypertension and LA volume and function (4, 6). Ikejder et al. (4) conducted a cross-sectional investigation on the evaluation of the relationship between hypertension and LA volume and function. Using two-dimensional echocardiography, the investigation showed increases in LA reservoir function and pump function, while the LA conduit function decreased. However, some studies do not fully corroborate this result. Cioffi et al. (26) showed that atrial phasic changes in untreated individuals with severe hypertension demonstrated a decrease in conduit function and passive volume concurrent with an increase in the active emptying function. Another study in individuals with mild hypertension showed no change in the passive emptying function or conduit volumes, with an increase in the active emptying volume and an overall increase in LA volume, compared to people without hypertension (27).

Although there are few left atrium studies, there are many studies on hypertension and the left ventricle (28). However, there has been little research on how genetics can play a role here. Hypertension-induced remodeling of the LV includes concentric remodeling, concentric LV hypertrophy and eccentric LVH (29). Both LVM and LVMVR, the indexes that assess LV concentricity, are strong predictors of incipient cardiovascular events (30). The current study found they were positively affected by hypertension. In addition, the left ventricle’s structure was positively affected by hypertension. Our study provides evidence for a causal relationship between hypertension and LV structure and function on a genetic level. Similar associations between hypertension and LV function have previously been reported (31). The change in LV remodeling may be a major cause, as our study determined. The LV mechanical function involves a complex interaction between cardiomyocytes and non-myocytes of the heart, such as endothelial cells, fibroblasts and the immune system (32). Upregulation of the sympathetic nervous system is another important pathophysiological mechanism (33) to consider.

Clinically, it is often found that LA changes precede LV hypertrophy in hypertensive patients (34–38). While all studies are based on clinical and not supported by basic research. By now, the mechanism by which hypertension causes LA enlargement first is still under investigation. One explanation is that the increase in LA size may be a mechanism for counterbalancing the impairment of LV compliance and progression of LV diastolic dysfunction in the hypertrophied ventricle. LA enlargement usually develops well before hypertensive LV hypertrophy (LVH) manifests (38). Nevertheless, careful monitoring of LA/LV structure and function may play a role as a surrogate marker to monitor the efficacy of hypertension therapy.

This study has several strengths. One of the key strengths was the absence of reverse causality and the minimization of residual confoundment. Another strength is that the data we used came from large-scale GWASs, thereby providing sufficient sample sizes for strong statistical power. This study also has several limitations. First is the pleiotropy, which includes vertical pleiotropy and horizontal pleiotropy. Vertical pleiotropy is when the SNP influences one trait (exposure), which in turn influences another (outcome). Horizontal pleiotropy is when the SNP independently influences two traits. Vertical pleiotropy can be tested using MR analysis, whereas horizontal pleiotropy should be avoided in MR. It is hard to prove that the vertical pleiotropy mediated by the exposure cannot be biased due to SNPs that influence the two traits through independent pathways. Thus, we applied two main methods to detect horizontal pleiotropy, including the MR-Egger intercept and MR-PRESSO, hoping to minimize the bias it caused. Second, due to limited data on outcome variables, we found limited data on the atrium and ventricle in the current database, making validation of the outcome impossible. However, the data volume of the atrioventricular ventricle in this study was large enough to narrow the bias from the side.

To avoid the effects of population stratification, our findings are limited to individuals of European ancestry, and the extrapolation to other ethnic groups is not clear. Further studies of hypertension and LA/LV structure and function are needed. Secondly, there is no analysis of gene-environment interaction in this study. A further study of the correlation between hypertension and LA/LV structure and function could clarify this issue.

## Conclusion

In summary, the present study identified evidence that supports a potential causal link between hypertension and LA/LV structure and function.

## Data availability statement

The original contributions presented in this study are included in the article/Supplementary material, further inquiries can be directed to the corresponding authors.

## Author contributions

YJ, WS, and YS contributed to the conception and design of the study. YS organized the database and wrote the first draft of the manuscript. YZ performed the statistical analysis. NX, CB, and XL wrote sections of the manuscript. All authors contributed to manuscript revision, read, and approved the submitted version.

## Footnotes

1
https://www.finngen.fi/en

2
http://www.phenoscanner.medschl.cam.ac.uk/
