# Supplementary material for: Assessing the causal role of hypertension on left atrial and left ventricular structure and function: A two-sample Mendelian randomization study
Source: Front Cardiovasc Med. 2022 Nov 2;9:1006380. doi: 10.3389/fcvm.2022.1006380 (PMC9666890; doi:10.3389/fcvm.2022.1006380)
Supplement: Supplementary file 1 [file Data_Sheet_1.docx]

Supplementary tables and figures

**Table S1** Information on instrumental variables of hypertension and LAmax.

| SNP | Chr | Hg19 Position | Allele | Nearby Gene | Hypertension | | | | LAmax | | | | F | R^2^ |
| --- | --- | --- | --- | --- | --- | --- | --- | --- | --- | --- | --- | --- | --- | --- |
|  |  |  |  |  | BETA | SE | P | N | BETA | SE | P | N |  |  |
| rs10206833 | 2 | 159462894 | A/G | PKP4 | -0.079 | 0.0134 | 3.56E-09 | 218754 | -0.00184422 | 0.0131492 | 0.86 | 35648 | 34.75686645 | 0.000158862 |
| rs10217559 | 9 | 112751571 | C/T | PALM2-AKAP2 | 0.0584 | 0.0099 | 3.87E-09 | 218754 | -0.0044752 | 0.0080205 | 0.76 | 35648 | 34.79776368 | 0.000159049 |
| rs11065837 | 12 | 111684253 | A/G | CUX2 | -0.0721 | 0.0108 | 2.49E-11 | 218754 | 0.00732833 | 0.00886007 | 0.39 | 35648 | 44.5675795 | 0.000203694 |
| rs11564041 | 7 | 27324368 | C/T | LOC392008 | -0.1003 | 0.0149 | 1.52E-11 | 218754 | -0.0241382 | 0.0161094 | 0.15 | 35648 | 45.31326528 | 0.000207102 |
| rs11636952 | 15 | 75114322 | C/T | LMAN1L | -0.055 | 0.0094 | 4.21E-09 | 218754 | 0.00403954 | 0.00815134 | 0.62 | 35648 | 34.23463494 | 0.000156475 |
| rs11672460 | 19 | 2588423 | A/C | GNG7 | -0.168 | 0.0244 | 6.35E-12 | 218754 | -0.0730272 | 0.0305102 | 0.015 | 35648 | 47.4061777 | 0.000216665 |
| rs117627418 | 10 | 107370555 | C/T | N/A | 0.3041 | 0.0542 | 1.97E-08 | 218754 | 0.0832772 | 0.0419347 | 0.035 | 35648 | 31.4796791 | 0.000143885 |
| rs11773825 | 7 | 150075576 | A/C | REPIN1\|\|ZNF775 | -0.0627 | 0.0104 | 1.37E-09 | 218754 | -0.0028957 | 0.00848236 | 0.76 | 35648 | 36.34665364 | 0.000166127 |
| rs12567136 | 1 | 11883731 | C/T | CLCN6 | -0.1242 | 0.013 | 1.22E-21 | 218754 | -0.00208799 | 0.0100051 | 0.69 | 35648 | 91.27514182 | 0.00041708 |
| rs1275984 | 2 | 26911509 | A/C | CIB4\|\|KCNK3 | -0.0953 | 0.0092 | 3.38E-25 | 218754 | -0.012144 | 0.00768398 | 0.15 | 35648 | 107.3015946 | 0.000490277 |
| rs12828438 | 12 | 12883570 | A/G | CDKN1B\|\|MIRN613 | -0.0606 | 0.0092 | 5.01E-11 | 218754 | -0.00808208 | 0.00761175 | 0.34 | 35648 | 43.38759954 | 0.000198302 |
| rs13112725 | 4 | 106911742 | C/G | NPNT\|\|MGC16169 | 0.0697 | 0.0121 | 9.34E-09 | 218754 | 0.015192 | 0.00866429 | 0.083 | 35648 | 33.18110501 | 0.000151661 |
| rs1317181 | 1 | 230873488 | T/G | AGT\|\|CAPN9 | 0.0701 | 0.0112 | 4.04E-10 | 218754 | 0.0133734 | 0.00903924 | 0.16 | 35648 | 39.1738287 | 0.000179047 |
| rs1374264 | 2 | 164999883 | C/A | FIGN\|\|LOC100129745 | -0.0603 | 0.0092 | 6.95E-11 | 218754 | -0.0103512 | 0.00747538 | 0.18 | 35648 | 42.95908266 | 0.000196344 |
| rs143936272 | 17 | 57071147 | C/T | N/A | -0.1471 | 0.0264 | 2.61E-08 | 218754 | -0.0237917 | 0.0331081 | 0.42 | 35648 | 31.04656245 | 0.000141906 |
| rs145153053 | 17 | 45138033 | A/G | N/A | 0.0707 | 0.0115 | 7.54E-10 | 218754 | -0.000328241 | 0.00994394 | 0.95 | 35648 | 37.79542004 | 0.000172748 |
| rs145158522 | 10 | 106629859 | C/T | N/A | 0.2952 | 0.0523 | 1.63E-08 | 218754 | 0.0862624 | 0.0339284 | 0.0073 | 35648 | 31.85850249 | 0.000145616 |
| rs149150643 | 4 | 185877562 | C/T | N/A | -0.1328 | 0.0222 | 2.27E-09 | 218754 | -0.0470121 | 0.0174202 | 0.015 | 35648 | 35.78378127 | 0.000163555 |
| rs16853076 | 3 | 168774754 | C/T | C3orf50\|\|EVI1 | -0.1096 | 0.0192 | 1.06E-08 | 218754 | -0.0159642 | 0.0143424 | 0.19 | 35648 | 32.58477153 | 0.000148935 |
| rs181364994 | 20 | 31011152 | C/T | N/A | 0.1927 | 0.0332 | 6.53E-09 | 218754 | -0.0153667 | 0.0462486 | 0.69 | 35648 | 33.68862544 | 0.00015398 |
| rs183834802 | 19 | 11662022 | A/G | N/A | -0.2859 | 0.0446 | 1.45E-10 | 218754 | -0.0387698 | 0.0417063 | 0.44 | 35648 | 41.09174862 | 0.000187811 |
| rs1888693 | 10 | 18440444 | G/A | CACNB2 | 0.06 | 0.0094 | 1.45E-10 | 218754 | 0.00617342 | 0.00785373 | 0.42 | 35648 | 40.74204489 | 0.000186213 |
| rs1894400 | 15 | 91428955 | C/T | FES | 0.0834 | 0.0103 | 4.97E-16 | 218754 | 0.00918258 | 0.00798389 | 0.22 | 35648 | 65.5622246 | 0.00029962 |
| rs193255470 | 17 | 56137420 | C/T | N/A | -0.1661 | 0.0276 | 1.81E-09 | 218754 | 0.0148547 | 0.031177 | 0.64 | 35648 | 36.21738836 | 0.000165536 |
| rs1980235 | 12 | 90110782 | G/A | LOC643153\|\|MRPL2P1 | 0.0663 | 0.0106 | 4.59E-10 | 218754 | 0.00265137 | 0.00796513 | 0.7 | 35648 | 39.12112684 | 0.000178806 |
| rs198833 | 6 | 26114508 | A/G | HIST1H1T\|\|HIST1H2BC | -0.0885 | 0.0146 | 1.19E-09 | 218754 | -0.00341444 | 0.0102836 | 0.78 | 35648 | 36.74319006 | 0.000167939 |
| rs2274224 | 10 | 96039597 | C/G | PLCE1/LOC100128054 | -0.0751 | 0.0096 | 5.64E-15 | 218754 | -0.0162779 | 0.0075044 | 0.034 | 35648 | 61.19746566 | 0.000279679 |
| rs2392929 | 7 | 106414069 | G/T | FLJ36031\|\|PIK3CG | 0.062 | 0.01 | 5.21E-10 | 218754 | 0.0242434 | 0.00934317 | 0.0076 | 35648 | 38.43964855 | 0.000175692 |
| rs2643826 | 3 | 27562988 | C/T | SLC4A7\|\|LOC643634 | 0.0627 | 0.0093 | 1.75E-11 | 218754 | 0.0103397 | 0.00749235 | 0.26 | 35648 | 45.4532785 | 0.000207741 |
| rs2704368 | 2 | 164443803 | A/G | KCNH7\|\|FIGN | -0.0802 | 0.0124 | 9.52E-11 | 218754 | -0.0125004 | 0.0123907 | 0.24 | 35648 | 41.83130329 | 0.000191191 |
| rs2782981 | 10 | 115781547 | C/T | NHLRC2\|\|LOC100132839 | 0.0799 | 0.0096 | 8.71E-17 | 218754 | -0.00759767 | 0.0083172 | 0.35 | 35648 | 69.27030852 | 0.000316561 |
| rs35427 | 12 | 115556307 | T/G | TBX3\|\|LOC100129020 | -0.0592 | 0.0096 | 5.91E-10 | 218754 | -0.0242182 | 0.00781745 | 0.002 | 35648 | 38.0274301 | 0.000173808 |
| rs35619711 | 17 | 59482169 | -/C | TBX2 | 0.0614 | 0.0102 | 1.65E-09 | 218754 | 0.00221574 | 0.00841346 | 0.77 | 35648 | 36.23534729 | 0.000165618 |
| rs3790604 | 1 | 113046879 | A/C | WNT2B | 0.1321 | 0.0121 | 1.40E-27 | 218754 | 0.0273156 | 0.0142242 | 0.058 | 35648 | 119.1875586 | 0.000544556 |
| rs3796585 | 4 | 156639174 | G/A | GUCY1A3 | -0.0751 | 0.0096 | 4.48E-15 | 218754 | 0.000313253 | 0.00768185 | 0.86 | 35648 | 61.19746566 | 0.000279679 |
| rs4371736 | 5 | 157877453 | C/G | LOC100130177\|\|EBF1 | -0.0527 | 0.0094 | 2.09E-08 | 218754 | -0.0139712 | 0.00759995 | 0.068 | 35648 | 31.43124274 | 0.000143664 |
| rs4685218 | 3 | 14894140 | C/T | FGD5 | 0.0984 | 0.0153 | 1.30E-10 | 218754 | 0.00192053 | 0.0130184 | 0.76 | 35648 | 41.3621747 | 0.000189047 |
| rs557675 | 11 | 65566719 | T/G | OVOL1\|\|SNX32 | -0.0613 | 0.0093 | 5.42E-11 | 218754 | 0.00219172 | 0.0074823 | 0.75 | 35648 | 43.44612839 | 0.00019857 |
| rs62426324 | 6 | 127142458 | C/T | LOC442257\|\|RSPO3 | 0.0528 | 0.0091 | 7.13E-09 | 218754 | 0.0257247 | 0.00750997 | 0.0012 | 35648 | 33.66519154 | 0.000153873 |
| rs62434119 | 6 | 150984431 | C/T | PLEKHG1 | -0.1345 | 0.0172 | 6.23E-15 | 218754 | -0.0248588 | 0.0143711 | 0.091 | 35648 | 61.14820378 | 0.000279454 |
| rs62455829 | 7 | 70045931 | A/G | AUTS2 | -0.0598 | 0.0103 | 5.68E-09 | 218754 | 0.0143694 | 0.00957778 | 0.14 | 35648 | 33.70729857 | 0.000154065 |
| rs6668768 | 1 | 25136203 | C/T | CLIC4 | 0.0668 | 0.0111 | 1.87E-09 | 218754 | 0.0142804 | 0.0111261 | 0.16 | 35648 | 36.21620975 | 0.000165531 |
| rs6860901 | 5 | 127871750 | C/T | FBN2 | 0.0682 | 0.01 | 7.68E-12 | 218754 | 0.0151168 | 0.00806814 | 0.049 | 35648 | 46.51197475 | 0.000212579 |
| rs6918791 | 6 | 126218961 | C/G | NCOA7 | 0.0574 | 0.0103 | 2.15E-08 | 218754 | -0.00704786 | 0.00836778 | 0.37 | 35648 | 31.05598904 | 0.000141949 |
| rs7123467 | 11 | 10155174 | C/G | SBF2 | 0.0682 | 0.0114 | 2.47E-09 | 218754 | 0.0014549 | 0.0113763 | 0.87 | 35648 | 35.78945426 | 0.000163581 |
| rs7134677 | 12 | 54441498 | C/T | HOXC4 | -0.0748 | 0.0094 | 2.13E-15 | 218754 | -0.0142652 | 0.00818986 | 0.11 | 35648 | 63.32038079 | 0.000289378 |
| rs72989964 | 19 | 12428623 | C/T | N/A | -0.248 | 0.0443 | 2.14E-08 | 218754 | -0.0621758 | 0.0456247 | 0.18 | 35648 | 31.339491 | 0.000143244 |
| rs7483477 | 11 | 1920255 | T/G | LSP1\|\|TNNT3 | 0.0759 | 0.0109 | 3.12E-12 | 218754 | 0.0171546 | 0.00868581 | 0.039 | 35648 | 48.48714191 | 0.000221604 |
| rs7545442 | 1 | 27260783 | C/T | NUDC | 0.098 | 0.0176 | 2.74E-08 | 218754 | 0.00500875 | 0.0140381 | 0.7 | 35648 | 31.00436529 | 0.000141713 |
| rs78302204 | 20 | 57735448 | A/G | N/A | 0.1406 | 0.0122 | 1.03E-30 | 218754 | 0.0236856 | 0.01213 | 0.05 | 35648 | 132.8149641 | 0.00060678 |
| rs79873333 | 17 | 47357173 | C/T | N/A | -0.1364 | 0.0215 | 2.11E-10 | 218754 | -0.00772383 | 0.0218607 | 0.61 | 35648 | 40.24832861 | 0.000183957 |
| rs880315 | 1 | 10796866 | T/C | CASZ1 | 0.1028 | 0.0093 | 1.83E-28 | 218754 | 0.023143 | 0.00792581 | 0.0061 | 35648 | 122.1845691 | 0.000558241 |
| rs9899012 | 17 | 61545486 | A/G | CYB561\|\|LOC342541 | -0.1068 | 0.0166 | 1.41E-10 | 218754 | 0.00154509 | 0.0158389 | 0.99 | 35648 | 41.39256683 | 0.000189186 |

Note: Chr, chromosome; A1 and A2 are effect allele and alternative allele, respectively; SE, standard error; F, F statistics; LAmax, LA maximum volumes.

**Table S2** Information on instrumental variables of hypertension and LAmin.

| SNP | Chr | Hg19 Position | Allele | Nearby Gene | Hypertension | | | | LAmin | | | | F | R^2^ |
| --- | --- | --- | --- | --- | --- | --- | --- | --- | --- | --- | --- | --- | --- | --- |
|  |  |  |  |  | BETA | SE | P | N | BETA | SE | P | N |  |  |
| rs10206833 | 2 | 159462894 | A/G | PKP4 | -0.079 | 0.0134 | 3.56E-09 | 218754 | -0.00157373 | 0.0131694 | 0.85 | 35648 | 34.75686645 | 0.000158862 |
| rs10217559 | 9 | 112751571 | C/T | PALM2-AKAP2 | 0.0584 | 0.0099 | 3.87E-09 | 218754 | -0.0027257 | 0.00803283 | 0.89 | 35648 | 34.79776368 | 0.000159049 |
| rs11065837 | 12 | 111684253 | A/G | CUX2 | -0.0721 | 0.0108 | 2.49E-11 | 218754 | 0.00420442 | 0.00887422 | 0.6 | 35648 | 44.5675795 | 0.000203694 |
| rs11564041 | 7 | 27324368 | C/T | LOC392008 | -0.1003 | 0.0149 | 1.52E-11 | 218754 | -0.0384681 | 0.0161306 | 0.024 | 35648 | 45.31326528 | 0.000207102 |
| rs11636952 | 15 | 75114322 | C/T | LMAN1L | -0.055 | 0.0094 | 4.21E-09 | 218754 | -0.000271003 | 0.00816292 | 0.98 | 35648 | 34.23463494 | 0.000156475 |
| rs11672460 | 19 | 2588423 | A/C | GNG7 | -0.168 | 0.0244 | 6.35E-12 | 218754 | -0.0386828 | 0.0305552 | 0.2 | 35648 | 47.4061777 | 0.000216665 |
| rs117627418 | 10 | 107370555 | C/T | N/A | 0.3041 | 0.0542 | 1.97E-08 | 218754 | 0.0728453 | 0.041996 | 0.063 | 35648 | 31.4796791 | 0.000143885 |
| rs11773825 | 7 | 150075576 | A/C | REPIN1\|\|ZNF775 | -0.0627 | 0.0104 | 1.37E-09 | 218754 | -0.0087615 | 0.00849352 | 0.32 | 35648 | 36.34665364 | 0.000166127 |
| rs12567136 | 1 | 11883731 | C/T | CLCN6 | -0.1242 | 0.013 | 1.22E-21 | 218754 | 0.0057959 | 0.0100209 | 0.73 | 35648 | 91.27514182 | 0.00041708 |
| rs1275984 | 2 | 26911509 | A/C | CIB4\|\|KCNK3 | -0.0953 | 0.0092 | 3.38E-25 | 218754 | -0.00579141 | 0.00769577 | 0.53 | 35648 | 107.3015946 | 0.000490277 |
| rs12828438 | 12 | 12883570 | A/G | CDKN1B\|\|MIRN613 | -0.0606 | 0.0092 | 5.01E-11 | 218754 | -0.0068331 | 0.0076239 | 0.42 | 35648 | 43.38759954 | 0.000198302 |
| rs13112725 | 4 | 106911742 | C/G | NPNT\|\|MGC16169 | 0.0697 | 0.0121 | 9.34E-09 | 218754 | 0.0159417 | 0.00867835 | 0.072 | 35648 | 33.18110501 | 0.000151661 |
| rs1317181 | 1 | 230873488 | T/G | AGT\|\|CAPN9 | 0.0701 | 0.0112 | 4.04E-10 | 218754 | 0.0138899 | 0.00905357 | 0.14 | 35648 | 39.1738287 | 0.000179047 |
| rs1374264 | 2 | 164999883 | C/A | FIGN\|\|LOC100129745 | -0.0603 | 0.0092 | 6.95E-11 | 218754 | -0.0136171 | 0.00748685 | 0.076 | 35648 | 42.95908266 | 0.000196344 |
| rs143936272 | 17 | 57071147 | C/T | N/A | -0.1471 | 0.0264 | 2.61E-08 | 218754 | -0.027932 | 0.0331591 | 0.36 | 35648 | 31.04656245 | 0.000141906 |
| rs145153053 | 17 | 45138033 | A/G | N/A | 0.0707 | 0.0115 | 7.54E-10 | 218754 | 0.00691116 | 0.00995927 | 0.47 | 35648 | 37.79542004 | 0.000172748 |
| rs145158522 | 10 | 106629859 | C/T | N/A | 0.2952 | 0.0523 | 1.63E-08 | 218754 | 0.0767079 | 0.033978 | 0.016 | 35648 | 31.85850249 | 0.000145616 |
| rs149150643 | 4 | 185877562 | C/T | N/A | -0.1328 | 0.0222 | 2.27E-09 | 218754 | -0.0510303 | 0.0174485 | 0.0074 | 35648 | 35.78378127 | 0.000163555 |
| rs16853076 | 3 | 168774754 | C/T | C3orf50\|\|EVI1 | -0.1096 | 0.0192 | 1.06E-08 | 218754 | -0.0153421 | 0.0143668 | 0.22 | 35648 | 32.58477153 | 0.000148935 |
| rs181364994 | 20 | 31011152 | C/T | N/A | 0.1927 | 0.0332 | 6.53E-09 | 218754 | 0.00234843 | 0.0463099 | 0.96 | 35648 | 33.68862544 | 0.00015398 |
| rs183834802 | 19 | 11662022 | A/G | N/A | -0.2859 | 0.0446 | 1.45E-10 | 218754 | -0.0215106 | 0.0417678 | 0.71 | 35648 | 41.09174862 | 0.000187811 |
| rs1888693 | 10 | 18440444 | G/A | CACNB2 | 0.06 | 0.0094 | 1.45E-10 | 218754 | 0.00916846 | 0.00786521 | 0.26 | 35648 | 40.74204489 | 0.000186213 |
| rs1894400 | 15 | 91428955 | C/T | FES | 0.0834 | 0.0103 | 4.97E-16 | 218754 | 0.00972898 | 0.00799523 | 0.18 | 35648 | 65.5622246 | 0.00029962 |
| rs193255470 | 17 | 56137420 | C/T | N/A | -0.1661 | 0.0276 | 1.81E-09 | 218754 | -0.00567108 | 0.031225 | 0.87 | 35648 | 36.21738836 | 0.000165536 |
| rs1980235 | 12 | 90110782 | G/A | LOC643153\|\|MRPL2P1 | 0.0663 | 0.0106 | 4.59E-10 | 218754 | 0.00333882 | 0.00797785 | 0.61 | 35648 | 39.12112684 | 0.000178806 |
| rs198833 | 6 | 26114508 | A/G | HIST1H1T\|\|HIST1H2BC | -0.0885 | 0.0146 | 1.19E-09 | 218754 | -0.00848507 | 0.0102974 | 0.46 | 35648 | 36.74319006 | 0.000167939 |
| rs2274224 | 10 | 96039597 | C/G | PLCE1/LOC100128054 | -0.0751 | 0.0096 | 5.64E-15 | 218754 | -0.0103883 | 0.00751536 | 0.18 | 35648 | 61.19746566 | 0.000279679 |
| rs2392929 | 7 | 106414069 | G/T | FLJ36031\|\|PIK3CG | 0.062 | 0.01 | 5.21E-10 | 218754 | 0.0194392 | 0.00935545 | 0.029 | 35648 | 38.43964855 | 0.000175692 |
| rs2643826 | 3 | 27562988 | C/T | SLC4A7\|\|LOC643634 | 0.0627 | 0.0093 | 1.75E-11 | 218754 | 0.0149778 | 0.0075051 | 0.078 | 35648 | 45.4532785 | 0.000207741 |
| rs2704368 | 2 | 164443803 | A/G | KCNH7\|\|FIGN | -0.0802 | 0.0124 | 9.52E-11 | 218754 | -0.0199252 | 0.0124097 | 0.083 | 35648 | 41.83130329 | 0.000191191 |
| rs2782981 | 10 | 115781547 | C/T | NHLRC2\|\|LOC100132839 | 0.0799 | 0.0096 | 8.71E-17 | 218754 | -0.00562939 | 0.00832936 | 0.51 | 35648 | 69.27030852 | 0.000316561 |
| rs35427 | 12 | 115556307 | T/G | TBX3\|\|LOC100129020 | -0.0592 | 0.0096 | 5.91E-10 | 218754 | -0.0250756 | 0.00782994 | 0.0013 | 35648 | 38.0274301 | 0.000173808 |
| rs35619711 | 17 | 59482169 | -/C | TBX2 | 0.0614 | 0.0102 | 1.65E-09 | 218754 | -0.00862757 | 0.00842643 | 0.31 | 35648 | 36.23534729 | 0.000165618 |
| rs3790604 | 1 | 113046879 | A/C | WNT2B | 0.1321 | 0.0121 | 1.40E-27 | 218754 | 0.0290972 | 0.0142467 | 0.045 | 35648 | 119.1875586 | 0.000544556 |
| rs3796585 | 4 | 156639174 | G/A | GUCY1A3 | -0.0751 | 0.0096 | 4.48E-15 | 218754 | -0.00625652 | 0.00769431 | 0.49 | 35648 | 61.19746566 | 0.000279679 |
| rs4371736 | 5 | 157877453 | C/G | LOC100130177\|\|EBF1 | -0.0527 | 0.0094 | 2.09E-08 | 218754 | -0.0134233 | 0.00761152 | 0.071 | 35648 | 31.43124274 | 0.000143664 |
| rs4685218 | 3 | 14894140 | C/T | FGD5 | 0.0984 | 0.0153 | 1.30E-10 | 218754 | -0.00206282 | 0.0130406 | 0.97 | 35648 | 41.3621747 | 0.000189047 |
| rs557675 | 11 | 65566719 | T/G | OVOL1\|\|SNX32 | -0.0613 | 0.0093 | 5.42E-11 | 218754 | 0.000408433 | 0.00749461 | 0.98 | 35648 | 43.44612839 | 0.00019857 |
| rs62426324 | 6 | 127142458 | C/T | LOC442257\|\|RSPO3 | 0.0528 | 0.0091 | 7.13E-09 | 218754 | 0.0261536 | 0.0075201 | 0.00083 | 35648 | 33.66519154 | 0.000153873 |
| rs62434119 | 6 | 150984431 | C/T | PLEKHG1 | -0.1345 | 0.0172 | 6.23E-15 | 218754 | -0.00860214 | 0.0143905 | 0.61 | 35648 | 61.14820378 | 0.000279454 |
| rs62455829 | 7 | 70045931 | A/G | AUTS2 | -0.0598 | 0.0103 | 5.68E-09 | 218754 | 0.0131882 | 0.00959037 | 0.16 | 35648 | 33.70729857 | 0.000154065 |
| rs629042 | 13 | 22318506 | C/G | FGF9\|\|FTHL7 | 0.0595 | 0.0093 | 1.36E-10 | 218754 | 0.0212258 | 0.00765141 | 0.0025 | 35648 | 40.93210351 | 0.000187081 |
| rs6668768 | 1 | 25136203 | C/T | CLIC4 | 0.0668 | 0.0111 | 1.87E-09 | 218754 | 0.016645 | 0.0111437 | 0.11 | 35648 | 36.21620975 | 0.000165531 |
| rs6860901 | 5 | 127871750 | C/T | FBN2 | 0.0682 | 0.01 | 7.68E-12 | 218754 | 0.0133282 | 0.00808042 | 0.089 | 35648 | 46.51197475 | 0.000212579 |
| rs6918791 | 6 | 126218961 | C/G | NCOA7 | 0.0574 | 0.0103 | 2.15E-08 | 218754 | -0.00272823 | 0.00837907 | 0.73 | 35648 | 31.05598904 | 0.000141949 |
| rs7123467 | 11 | 10155174 | C/G | SBF2 | 0.0682 | 0.0114 | 2.47E-09 | 218754 | -0.00672306 | 0.0113951 | 0.58 | 35648 | 35.78945426 | 0.000163581 |
| rs7134677 | 12 | 54441498 | C/T | HOXC4 | -0.0748 | 0.0094 | 2.13E-15 | 218754 | -0.0060458 | 0.00820294 | 0.5 | 35648 | 63.32038079 | 0.000289378 |
| rs72989964 | 19 | 12428623 | C/T | N/A | -0.248 | 0.0443 | 2.14E-08 | 218754 | -0.0325754 | 0.0456919 | 0.48 | 35648 | 31.339491 | 0.000143244 |
| rs7483477 | 11 | 1920255 | T/G | LSP1\|\|TNNT3 | 0.0759 | 0.0109 | 3.12E-12 | 218754 | 0.0132387 | 0.00870011 | 0.11 | 35648 | 48.48714191 | 0.000221604 |
| rs7545442 | 1 | 27260783 | C/T | NUDC | 0.098 | 0.0176 | 2.74E-08 | 218754 | 0.00774594 | 0.0140604 | 0.56 | 35648 | 31.00436529 | 0.000141713 |
| rs78302204 | 20 | 57735448 | A/G | N/A | 0.1406 | 0.0122 | 1.03E-30 | 218754 | 0.0232464 | 0.012146 | 0.054 | 35648 | 132.8149641 | 0.00060678 |
| rs79873333 | 17 | 47357173 | C/T | N/A | -0.1364 | 0.0215 | 2.11E-10 | 218754 | 0.0151581 | 0.0218944 | 0.57 | 35648 | 40.24832861 | 0.000183957 |
| rs880315 | 1 | 10796866 | T/C | CASZ1 | 0.1028 | 0.0093 | 1.83E-28 | 218754 | 0.0198299 | 0.00793838 | 0.021 | 35648 | 122.1845691 | 0.000558241 |
| rs9899012 | 17 | 61545486 | A/G | CYB561\|\|LOC342541 | -0.1068 | 0.0166 | 1.41E-10 | 218754 | -0.000345267 | 0.0158633 | 0.97 | 35648 | 41.39256683 | 0.000189186 |

Note: Chr, chromosome; A1 and A2 are effect allele and alternative allele, respectively; SE, stantard error; F, F statistics; LAmin, LA minimum volumes.

**Table S3** Information on instrumental variables of hypertension and LAAEF.

| SNP | Chr | Hg19 Position | Allele | Nearby Gene | Hypertension | | | | LAAEF | | | | F | R^2^ |
| --- | --- | --- | --- | --- | --- | --- | --- | --- | --- | --- | --- | --- | --- | --- |
|  |  |  |  |  | BETA | SE | P | N | BETA | SE | P | N |  |  |
| rs10059884 | 5 | 32832474 | A/C | C5orf23\|\|LOC340113 | 0.0651 | 0.0092 | 1.92E-12 | 218754 | -0.0154473 | 0.00761855 | 0.043 | 35648 | 50.07054883 | 0.000228839 |
| rs10206833 | 2 | 159462894 | A/G | PKP4 | -0.079 | 0.0134 | 3.56E-09 | 218754 | 0.00850766 | 0.013193 | 0.46 | 35648 | 34.75686645 | 0.000158862 |
| rs10217559 | 9 | 112751571 | C/T | PALM2-AKAP2 | 0.0584 | 0.0099 | 3.87E-09 | 218754 | 0.000426601 | 0.00804682 | 0.98 | 35648 | 34.79776368 | 0.000159049 |
| rs11065837 | 12 | 111684253 | A/G | CUX2 | -0.0721 | 0.0108 | 2.49E-11 | 218754 | 0.000398913 | 0.00889091 | 0.98 | 35648 | 44.5675795 | 0.000203694 |
| rs11564041 | 7 | 27324368 | C/T | LOC392008 | -0.1003 | 0.0149 | 1.52E-11 | 218754 | 0.0361237 | 0.0161544 | 0.034 | 35648 | 45.31326528 | 0.000207102 |
| rs11636952 | 15 | 75114322 | C/T | LMAN1L | -0.055 | 0.0094 | 4.21E-09 | 218754 | 0.0068237 | 0.00817578 | 0.42 | 35648 | 34.23463494 | 0.000156475 |
| rs11672460 | 19 | 2588423 | A/C | GNG7 | -0.168 | 0.0244 | 6.35E-12 | 218754 | -0.0192205 | 0.0306035 | 0.57 | 35648 | 47.4061777 | 0.000216665 |
| rs117627418 | 10 | 107370555 | C/T | N/A | 0.3041 | 0.0542 | 1.97E-08 | 218754 | -0.0417432 | 0.0420643 | 0.3 | 35648 | 31.4796791 | 0.000143885 |
| rs11773825 | 7 | 150075576 | A/C | REPIN1\|\|ZNF775 | -0.0627 | 0.0104 | 1.37E-09 | 218754 | 0.00993094 | 0.00850605 | 0.29 | 35648 | 36.34665364 | 0.000166127 |
| rs12567136 | 1 | 11883731 | C/T | CLCN6 | -0.1242 | 0.013 | 1.22E-21 | 218754 | -0.0137474 | 0.0100397 | 0.24 | 35648 | 91.27514182 | 0.00041708 |
| rs1275984 | 2 | 26911509 | A/C | CIB4\|\|KCNK3 | -0.0953 | 0.0092 | 3.38E-25 | 218754 | -0.00984939 | 0.00770955 | 0.16 | 35648 | 107.3015946 | 0.000490277 |
| rs12828438 | 12 | 12883570 | A/G | CDKN1B\|\|MIRN613 | -0.0606 | 0.0092 | 5.01E-11 | 218754 | -0.00279623 | 0.00763824 | 0.72 | 35648 | 43.38759954 | 0.000198302 |
| rs13112725 | 4 | 106911742 | C/G | NPNT\|\|MGC16169 | 0.0697 | 0.0121 | 9.34E-09 | 218754 | -0.0209926 | 0.00869424 | 0.021 | 35648 | 33.18110501 | 0.000151661 |
| rs1317181 | 1 | 230873488 | T/G | AGT\|\|CAPN9 | 0.0701 | 0.0112 | 4.04E-10 | 218754 | -0.0158298 | 0.00907057 | 0.098 | 35648 | 39.1738287 | 0.000179047 |
| rs1374264 | 2 | 164999883 | C/A | FIGN\|\|LOC100129745 | -0.0603 | 0.0092 | 6.95E-11 | 218754 | 0.0145171 | 0.00750026 | 0.049 | 35648 | 42.95908266 | 0.000196344 |
| rs143936272 | 17 | 57071147 | C/T | N/A | -0.1471 | 0.0264 | 2.61E-08 | 218754 | 0.0281183 | 0.0332135 | 0.4 | 35648 | 31.04656245 | 0.000141906 |
| rs145153053 | 17 | 45138033 | A/G | N/A | 0.0707 | 0.0115 | 7.54E-10 | 218754 | 0.00566762 | 0.00997562 | 0.63 | 35648 | 37.79542004 | 0.000172748 |
| rs145158522 | 10 | 106629859 | C/T | N/A | 0.2952 | 0.0523 | 1.63E-08 | 218754 | -0.039405 | 0.0340333 | 0.21 | 35648 | 31.85850249 | 0.000145616 |
| rs149150643 | 4 | 185877562 | C/T | N/A | -0.1328 | 0.0222 | 2.27E-09 | 218754 | 0.0378121 | 0.0174804 | 0.042 | 35648 | 35.78378127 | 0.000163555 |
| rs16853076 | 3 | 168774754 | C/T | C3orf50\|\|EVI1 | -0.1096 | 0.0192 | 1.06E-08 | 218754 | -0.00423267 | 0.0143947 | 0.83 | 35648 | 32.58477153 | 0.000148935 |
| rs181364994 | 20 | 31011152 | C/T | N/A | 0.1927 | 0.0332 | 6.53E-09 | 218754 | -0.0185515 | 0.0463848 | 0.79 | 35648 | 33.68862544 | 0.00015398 |
| rs183834802 | 19 | 11662022 | A/G | N/A | -0.2859 | 0.0446 | 1.45E-10 | 218754 | -0.000374833 | 0.0418338 | 0.95 | 35648 | 41.09174862 | 0.000187811 |
| rs1888693 | 10 | 18440444 | G/A | CACNB2 | 0.06 | 0.0094 | 1.45E-10 | 218754 | -0.0115086 | 0.007878 | 0.19 | 35648 | 40.74204489 | 0.000186213 |
| rs1894400 | 15 | 91428955 | C/T | FES | 0.0834 | 0.0103 | 4.97E-16 | 218754 | -0.00282041 | 0.00800782 | 0.67 | 35648 | 65.5622246 | 0.00029962 |
| rs193255470 | 17 | 56137420 | C/T | N/A | -0.1661 | 0.0276 | 1.81E-09 | 218754 | 0.0227959 | 0.0312763 | 0.49 | 35648 | 36.21738836 | 0.000165536 |
| rs1980235 | 12 | 90110782 | G/A | LOC643153\|\|MRPL2P1 | 0.0663 | 0.0106 | 4.59E-10 | 218754 | -0.00191207 | 0.00799285 | 0.73 | 35648 | 39.12112684 | 0.000178806 |
| rs198833 | 6 | 26114508 | A/G | HIST1H1T\|\|HIST1H2BC | -0.0885 | 0.0146 | 1.19E-09 | 218754 | 0.000969312 | 0.0103157 | 0.92 | 35648 | 36.74319006 | 0.000167939 |
| rs2274224 | 10 | 96039597 | C/G | PLCE1/LOC100128054 | -0.0751 | 0.0096 | 5.64E-15 | 218754 | 0.00498184 | 0.00752759 | 0.47 | 35648 | 61.19746566 | 0.000279679 |
| rs2392929 | 7 | 106414069 | G/T | FLJ36031\|\|PIK3CG | 0.062 | 0.01 | 5.21E-10 | 218754 | -0.00428802 | 0.00936926 | 0.61 | 35648 | 38.43964855 | 0.000175692 |
| rs2643826 | 3 | 27562988 | C/T | SLC4A7\|\|LOC643634 | 0.0627 | 0.0093 | 1.75E-11 | 218754 | -0.0100184 | 0.00751966 | 0.22 | 35648 | 45.4532785 | 0.000207741 |
| rs2704368 | 2 | 164443803 | A/G | KCNH7\|\|FIGN | -0.0802 | 0.0124 | 9.52E-11 | 218754 | 0.0272929 | 0.0124319 | 0.022 | 35648 | 41.83130329 | 0.000191191 |
| rs2782981 | 10 | 115781547 | C/T | NHLRC2\|\|LOC100132839 | 0.0799 | 0.0096 | 8.71E-17 | 218754 | 0.00602552 | 0.00834291 | 0.47 | 35648 | 69.27030852 | 0.000316561 |
| rs35427 | 12 | 115556307 | T/G | TBX3\|\|LOC100129020 | -0.0592 | 0.0096 | 5.91E-10 | 218754 | 0.00782765 | 0.00784466 | 0.28 | 35648 | 38.0274301 | 0.000173808 |
| rs35619711 | 17 | 59482169 | -/C | TBX2 | 0.0614 | 0.0102 | 1.65E-09 | 218754 | 0.0149111 | 0.00844026 | 0.083 | 35648 | 36.23534729 | 0.000165618 |
| rs3790604 | 1 | 113046879 | A/C | WNT2B | 0.1321 | 0.0121 | 1.40E-27 | 218754 | -0.0154967 | 0.0142735 | 0.26 | 35648 | 119.1875586 | 0.000544556 |
| rs3796585 | 4 | 156639174 | G/A | GUCY1A3 | -0.0751 | 0.0096 | 4.48E-15 | 218754 | 0.00769639 | 0.0077084 | 0.34 | 35648 | 61.19746566 | 0.000279679 |
| rs4371736 | 5 | 157877453 | C/G | LOC100130177\|\|EBF1 | -0.0527 | 0.0094 | 2.09E-08 | 218754 | 0.00718668 | 0.00762623 | 0.29 | 35648 | 31.43124274 | 0.000143664 |
| rs4685218 | 3 | 14894140 | C/T | FGD5 | 0.0984 | 0.0153 | 1.30E-10 | 218754 | 0.0138114 | 0.0130659 | 0.36 | 35648 | 41.3621747 | 0.000189047 |
| rs557675 | 11 | 65566719 | T/G | OVOL1\|\|SNX32 | -0.0613 | 0.0093 | 5.42E-11 | 218754 | 0.00308396 | 0.0075074 | 0.6 | 35648 | 43.44612839 | 0.00019857 |
| rs62426324 | 6 | 127142458 | C/T | LOC442257\|\|RSPO3 | 0.0528 | 0.0091 | 7.13E-09 | 218754 | -0.0163745 | 0.00753341 | 0.033 | 35648 | 33.66519154 | 0.000153873 |
| rs62434119 | 6 | 150984431 | C/T | PLEKHG1 | -0.1345 | 0.0172 | 6.23E-15 | 218754 | -0.0122764 | 0.014416 | 0.33 | 35648 | 61.14820378 | 0.000279454 |
| rs62455829 | 7 | 70045931 | A/G | AUTS2 | -0.0598 | 0.0103 | 5.68E-09 | 218754 | -0.0134851 | 0.00960453 | 0.15 | 35648 | 33.70729857 | 0.000154065 |
| rs629042 | 13 | 22318506 | C/G | FGF9\|\|FTHL7 | 0.0595 | 0.0093 | 1.36E-10 | 218754 | -0.00828208 | 0.00766261 | 0.17 | 35648 | 40.93210351 | 0.000187081 |
| rs6668768 | 1 | 25136203 | C/T | CLIC4 | 0.0668 | 0.0111 | 1.87E-09 | 218754 | -0.0154146 | 0.0111647 | 0.15 | 35648 | 36.21620975 | 0.000165531 |
| rs6860901 | 5 | 127871750 | C/T | FBN2 | 0.0682 | 0.01 | 7.68E-12 | 218754 | -0.00601883 | 0.00809603 | 0.45 | 35648 | 46.51197475 | 0.000212579 |
| rs6918791 | 6 | 126218961 | C/G | NCOA7 | 0.0574 | 0.0103 | 2.15E-08 | 218754 | -0.00317593 | 0.0083939 | 0.67 | 35648 | 31.05598904 | 0.000141949 |
| rs7123467 | 11 | 10155174 | C/G | SBF2 | 0.0682 | 0.0114 | 2.47E-09 | 218754 | 0.0120362 | 0.0114145 | 0.32 | 35648 | 35.78945426 | 0.000163581 |
| rs7134677 | 12 | 54441498 | C/T | HOXC4 | -0.0748 | 0.0094 | 2.13E-15 | 218754 | -0.0119739 | 0.00821836 | 0.16 | 35648 | 63.32038079 | 0.000289378 |
| rs72989964 | 19 | 12428623 | C/T | N/A | -0.248 | 0.0443 | 2.14E-08 | 218754 | 0.00978453 | 0.0457642 | 0.8 | 35648 | 31.339491 | 0.000143244 |
| rs7483477 | 11 | 1920255 | T/G | LSP1\|\|TNNT3 | 0.0759 | 0.0109 | 3.12E-12 | 218754 | 0.00227942 | 0.00871496 | 0.8 | 35648 | 48.48714191 | 0.000221604 |
| rs7545442 | 1 | 27260783 | C/T | NUDC | 0.098 | 0.0176 | 2.74E-08 | 218754 | -0.006829 | 0.0140868 | 0.6 | 35648 | 31.00436529 | 0.000141713 |
| rs78302204 | 20 | 57735448 | A/G | N/A | 0.1406 | 0.0122 | 1.03E-30 | 218754 | -0.00845985 | 0.0121657 | 0.47 | 35648 | 132.8149641 | 0.00060678 |
| rs79873333 | 17 | 47357173 | C/T | N/A | -0.1364 | 0.0215 | 2.11E-10 | 218754 | -0.0497683 | 0.0219304 | 0.029 | 35648 | 40.24832861 | 0.000183957 |
| rs880315 | 1 | 10796866 | T/C | CASZ1 | 0.1028 | 0.0093 | 1.83E-28 | 218754 | -0.00140956 | 0.00795329 | 0.89 | 35648 | 122.1845691 | 0.000558241 |
| rs9899012 | 17 | 61545486 | A/G | CYB561\|\|LOC342541 | -0.1068 | 0.0166 | 1.41E-10 | 218754 | -0.0126091 | 0.0158893 | 0.42 | 35648 | 41.39256683 | 0.000189186 |

Note: Chr, chromosome; A1 and A2 are effect allele and alternative allele, respectively; SE, stantard error; F, F statistics; LAAEF, LA active emptying fraction.

**Table S4** Information on instrumental variables of hypertension and LAPEF.

| SNP | Chr | Hg19 Position | Allele | Nearby Gene | Hypertension | | | | LAPEF | | | | F | R^2^ |
| --- | --- | --- | --- | --- | --- | --- | --- | --- | --- | --- | --- | --- | --- | --- |
|  |  |  |  |  | BETA | SE | P | N | BETA | SE | P | N |  |  |
| rs10059884 | 5 | 32832474 | A/C | C5orf23\|\|LOC340113 | 0.0651 | 0.0092 | 1.92E-12 | 218754 | -0.0136106 | 0.00761395 | 0.086 | 35648 | 50.07054883 | 0.000228839 |
| rs10206833 | 2 | 159462894 | A/G | PKP4 | -0.079 | 0.0134 | 3.56E-09 | 218754 | -0.00440522 | 0.0131861 | 0.79 | 35648 | 34.75686645 | 0.000158862 |
| rs10217559 | 9 | 112751571 | C/T | PALM2-AKAP2 | 0.0584 | 0.0099 | 3.87E-09 | 218754 | 0.00298856 | 0.00804361 | 0.74 | 35648 | 34.79776368 | 0.000159049 |
| rs11065837 | 12 | 111684253 | A/G | CUX2 | -0.0721 | 0.0108 | 2.49E-11 | 218754 | 0.00149937 | 0.00888799 | 0.87 | 35648 | 44.5675795 | 0.000203694 |
| rs11564041 | 7 | 27324368 | C/T | LOC392008 | -0.1003 | 0.0149 | 1.52E-11 | 218754 | 0.0351819 | 0.0161463 | 0.036 | 35648 | 45.31326528 | 0.000207102 |
| rs11636952 | 15 | 75114322 | C/T | LMAN1L | -0.055 | 0.0094 | 4.21E-09 | 218754 | 0.00536754 | 0.00817462 | 0.48 | 35648 | 34.23463494 | 0.000156475 |
| rs11672460 | 19 | 2588423 | A/C | GNG7 | -0.168 | 0.0244 | 6.35E-12 | 218754 | 0.0237298 | 0.0305989 | 0.51 | 35648 | 47.4061777 | 0.000216665 |
| rs117627418 | 10 | 107370555 | C/T | N/A | 0.3041 | 0.0542 | 1.97E-08 | 218754 | -0.0202423 | 0.0420586 | 0.58 | 35648 | 31.4796791 | 0.000143885 |
| rs11773825 | 7 | 150075576 | A/C | REPIN1\|\|ZNF775 | -0.0627 | 0.0104 | 1.37E-09 | 218754 | 0.000197822 | 0.00850178 | 0.94 | 35648 | 36.34665364 | 0.000166127 |
| rs12567136 | 1 | 11883731 | C/T | CLCN6 | -0.1242 | 0.013 | 1.22E-21 | 218754 | -0.0129443 | 0.0100377 | 0.21 | 35648 | 91.27514182 | 0.00041708 |
| rs1275984 | 2 | 26911509 | A/C | CIB4\|\|KCNK3 | -0.0953 | 0.0092 | 3.38E-25 | 218754 | 0.0151247 | 0.00770553 | 0.049 | 35648 | 107.3015946 | 0.000490277 |
| rs12828438 | 12 | 12883570 | A/G | CDKN1B\|\|MIRN613 | -0.0606 | 0.0092 | 5.01E-11 | 218754 | 0.00615902 | 0.00763574 | 0.44 | 35648 | 43.38759954 | 0.000198302 |
| rs13112725 | 4 | 106911742 | C/G | NPNT\|\|MGC16169 | 0.0697 | 0.0121 | 9.34E-09 | 218754 | -0.00760216 | 0.00869046 | 0.37 | 35648 | 33.18110501 | 0.000151661 |
| rs1317181 | 1 | 230873488 | T/G | AGT\|\|CAPN9 | 0.0701 | 0.0112 | 4.04E-10 | 218754 | 0.00895044 | 0.00906871 | 0.4 | 35648 | 39.1738287 | 0.000179047 |
| rs1374264 | 2 | 164999883 | C/A | FIGN\|\|LOC100129745 | -0.0603 | 0.0092 | 6.95E-11 | 218754 | 0.00810554 | 0.00749634 | 0.3 | 35648 | 42.95908266 | 0.000196344 |
| rs143936272 | 17 | 57071147 | C/T | N/A | -0.1471 | 0.0264 | 2.61E-08 | 218754 | 0.0136963 | 0.0332084 | 0.69 | 35648 | 31.04656245 | 0.000141906 |
| rs145153053 | 17 | 45138033 | A/G | N/A | 0.0707 | 0.0115 | 7.54E-10 | 218754 | -0.00977293 | 0.00997407 | 0.34 | 35648 | 37.79542004 | 0.000172748 |
| rs145158522 | 10 | 106629859 | C/T | N/A | 0.2952 | 0.0523 | 1.63E-08 | 218754 | -0.0419869 | 0.0340286 | 0.24 | 35648 | 31.85850249 | 0.000145616 |
| rs149150643 | 4 | 185877562 | C/T | N/A | -0.1328 | 0.0222 | 2.27E-09 | 218754 | 0.0204724 | 0.0174728 | 0.24 | 35648 | 35.78378127 | 0.000163555 |
| rs16853076 | 3 | 168774754 | C/T | C3orf50\|\|EVI1 | -0.1096 | 0.0192 | 1.06E-08 | 218754 | 0.0345261 | 0.0143933 | 0.017 | 35648 | 32.58477153 | 0.000148935 |
| rs181364994 | 20 | 31011152 | C/T | N/A | 0.1927 | 0.0332 | 6.53E-09 | 218754 | -0.0260258 | 0.0463734 | 0.66 | 35648 | 33.68862544 | 0.00015398 |
| rs183834802 | 19 | 11662022 | A/G | N/A | -0.2859 | 0.0446 | 1.45E-10 | 218754 | 0.0080522 | 0.0418276 | 0.97 | 35648 | 41.09174862 | 0.000187811 |
| rs1888693 | 10 | 18440444 | G/A | CACNB2 | 0.06 | 0.0094 | 1.45E-10 | 218754 | -0.00367743 | 0.00787693 | 0.61 | 35648 | 40.74204489 | 0.000186213 |
| rs1894400 | 15 | 91428955 | C/T | FES | 0.0834 | 0.0103 | 4.97E-16 | 218754 | -0.0169156 | 0.00800669 | 0.027 | 35648 | 65.5622246 | 0.00029962 |
| rs193255470 | 17 | 56137420 | C/T | N/A | -0.1661 | 0.0276 | 1.81E-09 | 218754 | 0.0220244 | 0.0312714 | 0.51 | 35648 | 36.21738836 | 0.000165536 |
| rs1980235 | 12 | 90110782 | G/A | LOC643153\|\|MRPL2P1 | 0.0663 | 0.0106 | 4.59E-10 | 218754 | -0.0151194 | 0.00799024 | 0.051 | 35648 | 39.12112684 | 0.000178806 |
| rs198833 | 6 | 26114508 | A/G | HIST1H1T\|\|HIST1H2BC | -0.0885 | 0.0146 | 1.19E-09 | 218754 | 0.0212691 | 0.0103155 | 0.051 | 35648 | 36.74319006 | 0.000167939 |
| rs2274224 | 10 | 96039597 | C/G | PLCE1/LOC100128054 | -0.0751 | 0.0096 | 5.64E-15 | 218754 | -0.00519267 | 0.00752657 | 0.39 | 35648 | 61.19746566 | 0.000279679 |
| rs2392929 | 7 | 106414069 | G/T | FLJ36031\|\|PIK3CG | 0.062 | 0.01 | 5.21E-10 | 218754 | 0.00192775 | 0.00936456 | 0.92 | 35648 | 38.43964855 | 0.000175692 |
| rs2643826 | 3 | 27562988 | C/T | SLC4A7\|\|LOC643634 | 0.0627 | 0.0093 | 1.75E-11 | 218754 | -0.0184546 | 0.00751893 | 0.021 | 35648 | 45.4532785 | 0.000207741 |
| rs2704368 | 2 | 164443803 | A/G | KCNH7\|\|FIGN | -0.0802 | 0.0124 | 9.52E-11 | 218754 | 0.0170624 | 0.0124255 | 0.18 | 35648 | 41.83130329 | 0.000191191 |
| rs2782981 | 10 | 115781547 | C/T | NHLRC2\|\|LOC100132839 | 0.0799 | 0.0096 | 8.71E-17 | 218754 | -0.0112129 | 0.00834177 | 0.15 | 35648 | 69.27030852 | 0.000316561 |
| rs35427 | 12 | 115556307 | T/G | TBX3\|\|LOC100129020 | -0.0592 | 0.0096 | 5.91E-10 | 218754 | 0.0239231 | 0.00784209 | 0.0021 | 35648 | 38.0274301 | 0.000173808 |
| rs35619711 | 17 | 59482169 | -/C | TBX2 | 0.0614 | 0.0102 | 1.65E-09 | 218754 | 0.023918 | 0.00843895 | 0.0039 | 35648 | 36.23534729 | 0.000165618 |
| rs3790604 | 1 | 113046879 | A/C | WNT2B | 0.1321 | 0.0121 | 1.40E-27 | 218754 | -0.0254734 | 0.0142705 | 0.099 | 35648 | 119.1875586 | 0.000544556 |
| rs3796585 | 4 | 156639174 | G/A | GUCY1A3 | -0.0751 | 0.0096 | 4.48E-15 | 218754 | 0.00607534 | 0.00770505 | 0.49 | 35648 | 61.19746566 | 0.000279679 |
| rs4371736 | 5 | 157877453 | C/G | LOC100130177\|\|EBF1 | -0.0527 | 0.0094 | 2.09E-08 | 218754 | 0.016276 | 0.00762163 | 0.033 | 35648 | 31.43124274 | 0.000143664 |
| rs4685218 | 3 | 14894140 | C/T | FGD5 | 0.0984 | 0.0153 | 1.30E-10 | 218754 | -0.0130368 | 0.0130646 | 0.24 | 35648 | 41.3621747 | 0.000189047 |
| rs557675 | 11 | 65566719 | T/G | OVOL1\|\|SNX32 | -0.0613 | 0.0093 | 5.42E-11 | 218754 | -0.000201006 | 0.0075046 | 1 | 35648 | 43.44612839 | 0.00019857 |
| rs62426324 | 6 | 127142458 | C/T | LOC442257\|\|RSPO3 | 0.0528 | 0.0091 | 7.13E-09 | 218754 | -0.00697548 | 0.00753328 | 0.37 | 35648 | 33.66519154 | 0.000153873 |
| rs62434119 | 6 | 150984431 | C/T | PLEKHG1 | -0.1345 | 0.0172 | 6.23E-15 | 218754 | 0.00224251 | 0.0144157 | 0.94 | 35648 | 61.14820378 | 0.000279454 |
| rs62455829 | 7 | 70045931 | A/G | AUTS2 | -0.0598 | 0.0103 | 5.68E-09 | 218754 | 0.00352936 | 0.00959971 | 0.7 | 35648 | 33.70729857 | 0.000154065 |
| rs629042 | 13 | 22318506 | C/G | FGF9\|\|FTHL7 | 0.0595 | 0.0093 | 1.36E-10 | 218754 | -0.0034793 | 0.0076608 | 0.53 | 35648 | 40.93210351 | 0.000187081 |
| rs6668768 | 1 | 25136203 | C/T | CLIC4 | 0.0668 | 0.0111 | 1.87E-09 | 218754 | -0.0157938 | 0.0111624 | 0.17 | 35648 | 36.21620975 | 0.000165531 |
| rs6860901 | 5 | 127871750 | C/T | FBN2 | 0.0682 | 0.01 | 7.68E-12 | 218754 | -0.0111026 | 0.00809115 | 0.16 | 35648 | 46.51197475 | 0.000212579 |
| rs6918791 | 6 | 126218961 | C/G | NCOA7 | 0.0574 | 0.0103 | 2.15E-08 | 218754 | -0.00245734 | 0.00839375 | 0.78 | 35648 | 31.05598904 | 0.000141949 |
| rs7123467 | 11 | 10155174 | C/G | SBF2 | 0.0682 | 0.0114 | 2.47E-09 | 218754 | 0.00698183 | 0.0114102 | 0.49 | 35648 | 35.78945426 | 0.000163581 |
| rs7134677 | 12 | 54441498 | C/T | HOXC4 | -0.0748 | 0.0094 | 2.13E-15 | 218754 | 0.00976977 | 0.00821567 | 0.22 | 35648 | 63.32038079 | 0.000289378 |
| rs72989964 | 19 | 12428623 | C/T | N/A | -0.248 | 0.0443 | 2.14E-08 | 218754 | -0.0417914 | 0.0457573 | 0.28 | 35648 | 31.339491 | 0.000143244 |
| rs7483477 | 11 | 1920255 | T/G | LSP1\|\|TNNT3 | 0.0759 | 0.0109 | 3.12E-12 | 218754 | -0.0202085 | 0.0087117 | 0.017 | 35648 | 48.48714191 | 0.000221604 |
| rs7545442 | 1 | 27260783 | C/T | NUDC | 0.098 | 0.0176 | 2.74E-08 | 218754 | 0.00643571 | 0.0140839 | 0.72 | 35648 | 31.00436529 | 0.000141713 |
| rs78302204 | 20 | 57735448 | A/G | N/A | 0.1406 | 0.0122 | 1.03E-30 | 218754 | -0.0246506 | 0.0121627 | 0.039 | 35648 | 132.8149641 | 0.00060678 |
| rs79873333 | 17 | 47357173 | C/T | N/A | -0.1364 | 0.0215 | 2.11E-10 | 218754 | -0.0217786 | 0.0219269 | 0.34 | 35648 | 40.24832861 | 0.000183957 |
| rs880315 | 1 | 10796866 | T/C | CASZ1 | 0.1028 | 0.0093 | 1.83E-28 | 218754 | -0.0148187 | 0.00795165 | 0.11 | 35648 | 122.1845691 | 0.000558241 |
| rs9899012 | 17 | 61545486 | A/G | CYB561\|\|LOC342541 | -0.1068 | 0.0166 | 1.41E-10 | 218754 | 0.0382317 | 0.0158869 | 0.015 | 35648 | 41.39256683 | 0.000189186 |

Note: Chr, chromosome; A1 and A2 are effect allele and alternative allele, respectively; SE, stantard error; F, F statistics; LAPEF, LA passive emptying fraction.

**Table S5** Information on instrumental variables of hypertension and LATEF.

| SNP | Chr | Hg19 Position | Allele | Nearby Gene | Hypertension | | | | LATEF | | | | F | R^2^ |
| --- | --- | --- | --- | --- | --- | --- | --- | --- | --- | --- | --- | --- | --- | --- |
|  |  |  |  |  | BETA | SE | P | N | BETA | SE | P | N |  |  |
| rs10059884 | 5 | 32832474 | A/C | C5orf23\|\|LOC340113 | 0.0651 | 0.0092 | 1.92E-12 | 218754 | -0.0155165 | 0.00762165 | 0.039 | 35648 | 50.07054883 | 0.000228839 |
| rs10206833 | 2 | 159462894 | A/G | PKP4 | -0.079 | 0.0134 | 3.56E-09 | 218754 | 0.00328368 | 0.0132019 | 0.74 | 35648 | 34.75686645 | 0.000158862 |
| rs10217559 | 9 | 112751571 | C/T | PALM2-AKAP2 | 0.0584 | 0.0099 | 3.87E-09 | 218754 | 0.000197194 | 0.00805168 | 0.97 | 35648 | 34.79776368 | 0.000159049 |
| rs11065837 | 12 | 111684253 | A/G | CUX2 | -0.0721 | 0.0108 | 2.49E-11 | 218754 | 0.00123603 | 0.00889515 | 0.92 | 35648 | 44.5675795 | 0.000203694 |
| rs11564041 | 7 | 27324368 | C/T | LOC392008 | -0.1003 | 0.0149 | 1.52E-11 | 218754 | 0.0420378 | 0.016164 | 0.013 | 35648 | 45.31326528 | 0.000207102 |
| rs11636952 | 15 | 75114322 | C/T | LMAN1L | -0.055 | 0.0094 | 4.21E-09 | 218754 | 0.006656 | 0.00818096 | 0.42 | 35648 | 34.23463494 | 0.000156475 |
| rs11672460 | 19 | 2588423 | A/C | GNG7 | -0.168 | 0.0244 | 6.35E-12 | 218754 | -0.0227199 | 0.0306234 | 0.45 | 35648 | 47.4061777 | 0.000216665 |
| rs117627418 | 10 | 107370555 | -/- | N/A | 0.3041 | 0.0542 | 1.97E-08 | 218754 | -0.0368529 | 0.0420959 | 0.35 | 35648 | 31.4796791 | 0.000143885 |
| rs11773825 | 7 | 150075576 | A/C | REPIN1\|\|ZNF775 | -0.0627 | 0.0104 | 1.37E-09 | 218754 | 0.0134206 | 0.00851114 | 0.13 | 35648 | 36.34665364 | 0.000166127 |
| rs12567136 | 1 | 11883731 | C/T | CLCN6 | -0.1242 | 0.013 | 1.22E-21 | 218754 | -0.0139169 | 0.010047 | 0.23 | 35648 | 91.27514182 | 0.00041708 |
| rs1275984 | 2 | 26911509 | A/C | CIB4\|\|KCNK3 | -0.0953 | 0.0092 | 3.38E-25 | 218754 | -0.00531237 | 0.00771475 | 0.46 | 35648 | 107.3015946 | 0.000490277 |
| rs12828438 | 12 | 12883570 | A/G | CDKN1B\|\|MIRN613 | -0.0606 | 0.0092 | 5.01E-11 | 218754 | 0.00376044 | 0.00764189 | 0.65 | 35648 | 43.38759954 | 0.000198302 |
| rs13112725 | 4 | 106911742 | C/G | NPNT\|\|MGC16169 | 0.0697 | 0.0121 | 9.34E-09 | 218754 | -0.012031 | 0.00869941 | 0.18 | 35648 | 33.18110501 | 0.000151661 |
| rs1317181 | 1 | 230873488 | T/G | AGT\|\|CAPN9 | 0.0701 | 0.0112 | 4.04E-10 | 218754 | -0.00792134 | 0.00907713 | 0.38 | 35648 | 39.1738287 | 0.000179047 |
| rs1374264 | 2 | 164999883 | C/A | FIGN\|\|LOC100129745 | -0.0603 | 0.0092 | 6.95E-11 | 218754 | 0.0141785 | 0.00750532 | 0.061 | 35648 | 42.95908266 | 0.000196344 |
| rs143936272 | 17 | 57071147 | -/- | N/A | -0.1471 | 0.0264 | 2.61E-08 | 218754 | 0.0272348 | 0.0332355 | 0.4 | 35648 | 31.04656245 | 0.000141906 |
| rs145153053 | 17 | 45138033 | -/- | N/A | 0.0707 | 0.0115 | 7.54E-10 | 218754 | -0.0134348 | 0.00998223 | 0.16 | 35648 | 37.79542004 | 0.000172748 |
| rs145158522 | 10 | 106629859 | -/- | N/A | 0.2952 | 0.0523 | 1.63E-08 | 218754 | -0.0382778 | 0.0340589 | 0.23 | 35648 | 31.85850249 | 0.000145616 |
| rs149150643 | 4 | 185877562 | -/- | N/A | -0.1328 | 0.0222 | 2.27E-09 | 218754 | 0.038473 | 0.0174908 | 0.036 | 35648 | 35.78378127 | 0.000163555 |
| rs16853076 | 3 | 168774754 | C/T | C3orf50\|\|EVI1 | -0.1096 | 0.0192 | 1.06E-08 | 218754 | 0.0119367 | 0.0144042 | 0.38 | 35648 | 32.58477153 | 0.000148935 |
| rs181364994 | 20 | 31011152 | -/- | N/A | 0.1927 | 0.0332 | 6.53E-09 | 218754 | -0.0228064 | 0.0464119 | 0.69 | 35648 | 33.68862544 | 0.00015398 |
| rs183834802 | 19 | 11662022 | -/- | N/A | -0.2859 | 0.0446 | 1.45E-10 | 218754 | -0.0134415 | 0.041861 | 0.68 | 35648 | 41.09174862 | 0.000187811 |
| rs1888693 | 10 | 18440444 | G/A | CACNB2 | 0.06 | 0.0094 | 1.45E-10 | 218754 | -0.00873159 | 0.00788392 | 0.3 | 35648 | 40.74204489 | 0.000186213 |
| rs1894400 | 15 | 91428955 | C/T | FES | 0.0834 | 0.0103 | 4.97E-16 | 218754 | -0.00686546 | 0.0080129 | 0.34 | 35648 | 65.5622246 | 0.00029962 |
| rs193255470 | 17 | 56137420 | -/- | N/A | -0.1661 | 0.0276 | 1.81E-09 | 218754 | 0.0309976 | 0.031297 | 0.34 | 35648 | 36.21738836 | 0.000165536 |
| rs1980235 | 12 | 90110782 | G/A | LOC643153\|\|MRPL2P1 | 0.0663 | 0.0106 | 4.59E-10 | 218754 | -0.00436224 | 0.00799667 | 0.53 | 35648 | 39.12112684 | 0.000178806 |
| rs198833 | 6 | 26114508 | A/G | HIST1H1T\|\|HIST1H2BC | -0.0885 | 0.0146 | 1.19E-09 | 218754 | 0.0131185 | 0.0103222 | 0.24 | 35648 | 36.74319006 | 0.000167939 |
| rs2274224 | 10 | 96039597 | C/G | PLCE1/LOC100128054 | -0.0751 | 0.0096 | 5.64E-15 | 218754 | 0.000393805 | 0.00753325 | 0.95 | 35648 | 61.19746566 | 0.000279679 |
| rs2392929 | 7 | 106414069 | G/T | FLJ36031\|\|PIK3CG | 0.062 | 0.01 | 5.21E-10 | 218754 | -0.00506437 | 0.00937486 | 0.53 | 35648 | 38.43964855 | 0.000175692 |
| rs2643826 | 3 | 27562988 | C/T | SLC4A7\|\|LOC643634 | 0.0627 | 0.0093 | 1.75E-11 | 218754 | -0.0155542 | 0.00752464 | 0.052 | 35648 | 45.4532785 | 0.000207741 |
| rs2704368 | 2 | 164443803 | A/G | KCNH7\|\|FIGN | -0.0802 | 0.0124 | 9.52E-11 | 218754 | 0.026279 | 0.0124403 | 0.029 | 35648 | 41.83130329 | 0.000191191 |
| rs2782981 | 10 | 115781547 | C/T | NHLRC2\|\|LOC100132839 | 0.0799 | 0.0096 | 8.71E-17 | 218754 | 0.00139277 | 0.00834918 | 0.9 | 35648 | 69.27030852 | 0.000316561 |
| rs35427 | 12 | 115556307 | T/G | TBX3\|\|LOC100129020 | -0.0592 | 0.0096 | 5.91E-10 | 218754 | 0.0161881 | 0.00784841 | 0.035 | 35648 | 38.0274301 | 0.000173808 |
| rs35619711 | 17 | 59482169 | -/C | TBX2 | 0.0614 | 0.0102 | 1.65E-09 | 218754 | 0.0212169 | 0.00844586 | 0.011 | 35648 | 36.23534729 | 0.000165618 |
| rs3790604 | 1 | 113046879 | A/C | WNT2B | 0.1321 | 0.0121 | 1.40E-27 | 218754 | -0.0219553 | 0.0142838 | 0.13 | 35648 | 119.1875586 | 0.000544556 |
| rs3796585 | 4 | 156639174 | G/A | GUCY1A3 | -0.0751 | 0.0096 | 4.48E-15 | 218754 | 0.0125116 | 0.00771298 | 0.12 | 35648 | 61.19746566 | 0.000279679 |
| rs4371736 | 5 | 157877453 | C/G | LOC100130177\|\|EBF1 | -0.0527 | 0.0094 | 2.09E-08 | 218754 | 0.00921933 | 0.00762933 | 0.19 | 35648 | 31.43124274 | 0.000143664 |
| rs4685218 | 3 | 14894140 | C/T | FGD5 | 0.0984 | 0.0153 | 1.30E-10 | 218754 | 0.00688672 | 0.0130746 | 0.71 | 35648 | 41.3621747 | 0.000189047 |
| rs557675 | 11 | 65566719 | T/G | OVOL1\|\|SNX32 | -0.0613 | 0.0093 | 5.42E-11 | 218754 | 0.00359377 | 0.00751286 | 0.56 | 35648 | 43.44612839 | 0.00019857 |
| rs62426324 | 6 | 127142458 | C/T | LOC442257\|\|RSPO3 | 0.0528 | 0.0091 | 7.13E-09 | 218754 | -0.0186316 | 0.0075382 | 0.015 | 35648 | 33.66519154 | 0.000153873 |
| rs62434119 | 6 | 150984431 | C/T | PLEKHG1 | -0.1345 | 0.0172 | 6.23E-15 | 218754 | -0.0182801 | 0.0144251 | 0.18 | 35648 | 61.14820378 | 0.000279454 |
| rs62455829 | 7 | 70045931 | A/G | AUTS2 | -0.0598 | 0.0103 | 5.68E-09 | 218754 | -0.00746649 | 0.00961027 | 0.4 | 35648 | 33.70729857 | 0.000154065 |
| rs629042 | 13 | 22318506 | C/G | FGF9\|\|FTHL7 | 0.0595 | 0.0093 | 1.36E-10 | 218754 | -0.00848417 | 0.00766788 | 0.18 | 35648 | 40.93210351 | 0.000187081 |
| rs6668768 | 1 | 25136203 | C/T | CLIC4 | 0.0668 | 0.0111 | 1.87E-09 | 218754 | -0.0138057 | 0.0111727 | 0.21 | 35648 | 36.21620975 | 0.000165531 |
| rs6860901 | 5 | 127871750 | C/T | FBN2 | 0.0682 | 0.01 | 7.68E-12 | 218754 | -0.00629459 | 0.00809932 | 0.43 | 35648 | 46.51197475 | 0.000212579 |
| rs6918791 | 6 | 126218961 | C/G | NCOA7 | 0.0574 | 0.0103 | 2.15E-08 | 218754 | -0.00455571 | 0.00839924 | 0.57 | 35648 | 31.05598904 | 0.000141949 |
| rs7123467 | 11 | 10155174 | C/G | SBF2 | 0.0682 | 0.0114 | 2.47E-09 | 218754 | 0.0176106 | 0.0114228 | 0.13 | 35648 | 35.78945426 | 0.000163581 |
| rs7134677 | 12 | 54441498 | C/T | HOXC4 | -0.0748 | 0.0094 | 2.13E-15 | 218754 | -0.00615973 | 0.00822229 | 0.48 | 35648 | 63.32038079 | 0.000289378 |
| rs72989964 | 19 | 12428623 | -/- | N/A | -0.248 | 0.0443 | 2.14E-08 | 218754 | -0.0239543 | 0.0457939 | 0.6 | 35648 | 31.339491 | 0.000143244 |
| rs7483477 | 11 | 1920255 | T/G | LSP1\|\|TNNT3 | 0.0759 | 0.0109 | 3.12E-12 | 218754 | -0.00471149 | 0.00872129 | 0.57 | 35648 | 48.48714191 | 0.000221604 |
| rs7545442 | 1 | 27260783 | C/T | NUDC | 0.098 | 0.0176 | 2.74E-08 | 218754 | -0.011956 | 0.014097 | 0.39 | 35648 | 31.00436529 | 0.000141713 |
| rs78302204 | 20 | 57735448 | -/- | N/A | 0.1406 | 0.0122 | 1.03E-30 | 218754 | -0.0170867 | 0.0121728 | 0.16 | 35648 | 132.8149641 | 0.00060678 |
| rs79873333 | 17 | 47357173 | -/- | N/A | -0.1364 | 0.0215 | 2.11E-10 | 218754 | -0.0450794 | 0.0219449 | 0.045 | 35648 | 40.24832861 | 0.000183957 |
| rs880315 | 1 | 10796866 | T/C | CASZ1 | 0.1028 | 0.0093 | 1.83E-28 | 218754 | -0.00688717 | 0.00795903 | 0.46 | 35648 | 122.1845691 | 0.000558241 |
| rs9899012 | 17 | 61545486 | A/G | CYB561\|\|LOC342541 | -0.1068 | 0.0166 | 1.41E-10 | 218754 | 0.00233594 | 0.0158999 | 0.94 | 35648 | 41.39256683 | 0.000189186 |

Note: Chr, chromosome; A1 and A2 are effect allele and alternative allele, respectively; SE, stantard error; F, F statistics; LATEF, LA total emptying fraction.

**Table S6** Information on instrumental variables of hypertension and LVEDV.

| SNP | Chr | Hg19 Position | Allele | Nearby Gene | Hypertension | | | | LVEDV | | | | F | R^2^ |
| --- | --- | --- | --- | --- | --- | --- | --- | --- | --- | --- | --- | --- | --- | --- |
|  |  |  |  |  | BETA | SE | P | N | BETA | SE | P | N |  |  |
| rs10059884 | 5 | 32832474 | A/C | C5orf23\|\|LOC340113 | 0.0651 | 0.0092 | 1.92E-12 | 218754 | 0.0117037 | 0.0109591 | 0.29 | 16920 | 50.07054883 | 0.000228839 |
| rs10206833 | 2 | 159462894 | A/G | PKP4 | -0.079 | 0.0134 | 3.56E-09 | 218754 | 0.00825118 | 0.0189071 | 0.66 | 16920 | 34.75686645 | 0.000158862 |
| rs10217559 | 9 | 112751571 | C/T | PALM2-AKAP2 | 0.0584 | 0.0099 | 3.87E-09 | 218754 | 0.0131219 | 0.0116053 | 0.26 | 16920 | 34.79776368 | 0.000159049 |
| rs11065837 | 12 | 111684253 | A/G | CUX2 | -0.0721 | 0.0108 | 2.49E-11 | 218754 | 0.0353518 | 0.0128042 | 0.0058 | 16920 | 44.5675795 | 0.000203694 |
| rs11564041 | 7 | 27324368 | C/T | LOC392008 | -0.1003 | 0.0149 | 1.52E-11 | 218754 | -0.0337957 | 0.023456 | 0.15 | 16920 | 45.31326528 | 0.000207102 |
| rs11636952 | 15 | 75114322 | C/T | LMAN1L | -0.055 | 0.0094 | 4.21E-09 | 218754 | 0.0164173 | 0.0117929 | 0.16 | 16920 | 34.23463494 | 0.000156475 |
| rs11773825 | 7 | 150075576 | A/C | REPIN1\|\|ZNF775 | -0.0627 | 0.0104 | 1.37E-09 | 218754 | 0.0237886 | 0.0122558 | 0.052 | 16920 | 36.34665364 | 0.000166127 |
| rs12567136 | 1 | 11883731 | C/T | CLCN6 | -0.1242 | 0.013 | 1.22E-21 | 218754 | -0.0396178 | 0.0145023 | 0.0063 | 16920 | 91.27514182 | 0.00041708 |
| rs1275984 | 2 | 26911509 | A/C | CIB4\|\|KCNK3 | -0.0953 | 0.0092 | 3.38E-25 | 218754 | -0.0117421 | 0.0111026 | 0.29 | 16920 | 107.3015946 | 0.000490277 |
| rs12828438 | 12 | 12883570 | A/G | CDKN1B\|\|MIRN613 | -0.0606 | 0.0092 | 5.01E-11 | 218754 | -0.0162542 | 0.011023 | 0.14 | 16920 | 43.38759954 | 0.000198302 |
| rs13112725 | 4 | 106911742 | C/G | NPNT\|\|MGC16169 | 0.0697 | 0.0121 | 9.34E-09 | 218754 | 0.00442511 | 0.012462 | 0.72 | 16920 | 33.18110501 | 0.000151661 |
| rs1317181 | 1 | 230873488 | T/G | AGT\|\|CAPN9 | 0.0701 | 0.0112 | 4.04E-10 | 218754 | 0.0166366 | 0.0130642 | 0.2 | 16920 | 39.1738287 | 0.000179047 |
| rs1374264 | 2 | 164999883 | C/A | FIGN\|\|LOC100129745 | -0.0603 | 0.0092 | 6.95E-11 | 218754 | -0.025523 | 0.0108103 | 0.018 | 16920 | 42.95908266 | 0.000196344 |
| rs145153053 | 17 | 45138033 | A/G | N/A | 0.0707 | 0.0115 | 7.54E-10 | 218754 | 0.0373992 | 0.0143015 | 0.0089 | 16920 | 37.79542004 | 0.000172748 |
| rs149150643 | 4 | 185877562 | C/T | N/A | -0.1328 | 0.0222 | 2.27E-09 | 218754 | -0.0533042 | 0.0248546 | 0.032 | 16920 | 35.78378127 | 0.000163555 |
| rs16853076 | 3 | 168774754 | C/T | C3orf50\|\|EVI1 | -0.1096 | 0.0192 | 1.06E-08 | 218754 | 0.0084211 | 0.0205963 | 0.68 | 16920 | 32.58477153 | 0.000148935 |
| rs1888693 | 10 | 18440444 | G/A | CACNB2 | 0.06 | 0.0094 | 1.45E-10 | 218754 | -0.000827809 | 0.0113088 | 0.94 | 16920 | 40.74204489 | 0.000186213 |
| rs1894400 | 15 | 91428955 | C/T | FES | 0.0834 | 0.0103 | 4.97E-16 | 218754 | -0.0068307 | 0.011585 | 0.56 | 16920 | 65.5622246 | 0.00029962 |
| rs1980235 | 12 | 90110782 | G/A | LOC643153\|\|MRPL2P1 | 0.0663 | 0.0106 | 4.59E-10 | 218754 | 0.00408218 | 0.0115655 | 0.72 | 16920 | 39.12112684 | 0.000178806 |
| rs198833 | 6 | 26114508 | A/G | HIST1H1T\|\|HIST1H2BC | -0.0885 | 0.0146 | 1.19E-09 | 218754 | 0.0105267 | 0.0149601 | 0.48 | 16920 | 36.74319006 | 0.000167939 |
| rs2274224 | 10 | 96039597 | C/G | PLCE1/LOC100128054 | -0.0751 | 0.0096 | 5.64E-15 | 218754 | 0.0203932 | 0.0108877 | 0.061 | 16920 | 61.19746566 | 0.000279679 |
| rs2392929 | 7 | 106414069 | G/T | FLJ36031\|\|PIK3CG | 0.062 | 0.01 | 5.21E-10 | 218754 | 0.0234115 | 0.0135389 | 0.084 | 16920 | 38.43964855 | 0.000175692 |
| rs2643826 | 3 | 27562988 | C/T | SLC4A7\|\|LOC643634 | 0.0627 | 0.0093 | 1.75E-11 | 218754 | 0.00721525 | 0.0108645 | 0.51 | 16920 | 45.4532785 | 0.000207741 |
| rs2704368 | 2 | 164443803 | A/G | KCNH7\|\|FIGN | -0.0802 | 0.0124 | 9.52E-11 | 218754 | 0.00883036 | 0.0179099 | 0.62 | 16920 | 41.83130329 | 0.000191191 |
| rs2782981 | 10 | 115781547 | C/T | NHLRC2\|\|LOC100132839 | 0.0799 | 0.0096 | 8.71E-17 | 218754 | -0.00493872 | 0.0120274 | 0.68 | 16920 | 69.27030852 | 0.000316561 |
| rs28567725 | 16 | 53826028 | C/T | FTO | 0.0617 | 0.0093 | 2.76E-11 | 218754 | 0.0139212 | 0.0110005 | 0.21 | 16920 | 31.43124274 | 0.000143664 |
| rs35619711 | 17 | 59482169 | -/C | TBX2 | 0.0614 | 0.0102 | 1.65E-09 | 218754 | 0.0227998 | 0.0121837 | 0.061 | 16920 | 36.23534729 | 0.000165618 |
| rs3790604 | 1 | 113046879 | A/C | WNT2B | 0.1321 | 0.0121 | 1.40E-27 | 218754 | 0.023433 | 0.0205158 | 0.25 | 16920 | 119.1875586 | 0.000544556 |
| rs3796585 | 4 | 156639174 | G/A | GUCY1A3 | -0.0751 | 0.0096 | 4.48E-15 | 218754 | 0.0176151 | 0.0110848 | 0.11 | 16920 | 61.19746566 | 0.000279679 |
| rs4371736 | 5 | 157877453 | C/G | LOC100130177\|\|EBF1 | -0.0527 | 0.0094 | 2.09E-08 | 218754 | 0.0146116 | 0.0110137 | 0.18 | 16920 | 31.43124274 | 0.000143664 |
| rs4685218 | 3 | 14894140 | C/T | FGD5 | 0.0984 | 0.0153 | 1.30E-10 | 218754 | -0.032268 | 0.0187748 | 0.086 | 16920 | 41.3621747 | 0.000189047 |
| rs557675 | 11 | 65566719 | T/G | OVOL1\|\|SNX32 | -0.0613 | 0.0093 | 5.42E-11 | 218754 | 0.0178843 | 0.0108969 | 0.1 | 16920 | 43.44612839 | 0.00019857 |
| rs62426324 | 6 | 127142458 | C/T | LOC442257\|\|RSPO3 | 0.0528 | 0.0091 | 7.13E-09 | 218754 | -0.000511476 | 0.0108696 | 0.96 | 16920 | 33.66519154 | 0.000153873 |
| rs62434119 | 6 | 150984431 | C/T | PLEKHG1 | -0.1345 | 0.0172 | 6.23E-15 | 218754 | -0.0298621 | 0.0206968 | 0.15 | 16920 | 61.14820378 | 0.000279454 |
| rs62455829 | 7 | 70045931 | A/G | AUTS2 | -0.0598 | 0.0103 | 5.68E-09 | 218754 | 0.03103 | 0.013979 | 0.026 | 16920 | 33.70729857 | 0.000154065 |
| rs629042 | 13 | 22318506 | C/G | FGF9\|\|FTHL7 | 0.0595 | 0.0093 | 1.36E-10 | 218754 | 0.0231864 | 0.0109923 | 0.035 | 16920 | 40.93210351 | 0.000187081 |
| rs6668768 | 1 | 25136203 | C/T | CLIC4 | 0.0668 | 0.0111 | 1.87E-09 | 218754 | -0.0085557 | 0.0162645 | 0.6 | 16920 | 36.21620975 | 0.000165531 |
| rs6860901 | 5 | 127871750 | C/T | FBN2 | 0.0682 | 0.01 | 7.68E-12 | 218754 | 0.00931144 | 0.0116647 | 0.42 | 16920 | 46.51197475 | 0.000212579 |
| rs6918791 | 6 | 126218961 | C/G | NCOA7 | 0.0574 | 0.0103 | 2.15E-08 | 218754 | 0.0030847 | 0.012098 | 0.8 | 16920 | 31.05598904 | 0.000141949 |
| rs7123467 | 11 | 10155174 | C/G | SBF2 | 0.0682 | 0.0114 | 2.47E-09 | 218754 | 0.0273706 | 0.0165031 | 0.097 | 16920 | 35.78945426 | 0.000163581 |
| rs7134677 | 12 | 54441498 | C/T | HOXC4 | -0.0748 | 0.0094 | 2.13E-15 | 218754 | -0.0268734 | 0.0118399 | 0.023 | 16920 | 63.32038079 | 0.000289378 |
| rs7483477 | 11 | 1920255 | T/G | LSP1\|\|TNNT3 | 0.0759 | 0.0109 | 3.12E-12 | 218754 | 0.0236266 | 0.0125385 | 0.06 | 16920 | 48.48714191 | 0.000221604 |
| rs7545442 | 1 | 27260783 | C/T | NUDC | 0.098 | 0.0176 | 2.74E-08 | 218754 | 0.0393177 | 0.0202143 | 0.052 | 16920 | 31.00436529 | 0.000141713 |
| rs78302204 | 20 | 57735448 | A/G | N/A | 0.1406 | 0.0122 | 1.03E-30 | 218754 | 0.0165151 | 0.0175657 | 0.35 | 16920 | 132.8149641 | 0.00060678 |
| rs9899012 | 17 | 61545486 | A/G | CYB561\|\|LOC342541 | -0.1068 | 0.0166 | 1.41E-10 | 218754 | -0.0231241 | 0.0228126 | 0.31 | 16920 | 41.39256683 | 0.000189186 |

Note: Chr, chromosome; A1 and A2 are effect allele and alternative allele, respectively; SE, stantard error; F, F statistics; LV, left ventricular; LVEDV.

**Table S7** Information on instrumental variables of hypertension and LVESV.

| SNP | Chr | Hg19 Position | Allele | Nearby Gene | Hypertension | | | | LVESV | | | | F | R^2^ |
| --- | --- | --- | --- | --- | --- | --- | --- | --- | --- | --- | --- | --- | --- | --- |
|  |  |  |  |  | BETA | SE | P | N | BETA | SE | P | N |  |  |
| rs10059884 | 5 | 32832474 | A/C | C5orf23\|\|LOC340113 | 0.0651 | 0.0092 | 1.92E-12 | 218754 | 0.0175826 | 0.0109562 | 0.11 | 16920 | 50.07054883 | 0.000228839 |
| rs10206833 | 2 | 159462894 | A/G | PKP4 | -0.079 | 0.0134 | 3.56E-09 | 218754 | 0.00100789 | 0.0189043 | 0.96 | 16920 | 34.75686645 | 0.000158862 |
| rs10217559 | 9 | 112751571 | C/T | PALM2-AKAP2 | 0.0584 | 0.0099 | 3.87E-09 | 218754 | 0.0127828 | 0.0116054 | 0.27 | 16920 | 34.79776368 | 0.000159049 |
| rs11065837 | 12 | 111684253 | A/G | CUX2 | -0.0721 | 0.0108 | 2.49E-11 | 218754 | 0.042099 | 0.0128186 | 0.001 | 16920 | 44.5675795 | 0.000203694 |
| rs11564041 | 7 | 27324368 | C/T | LOC392008 | -0.1003 | 0.0149 | 1.52E-11 | 218754 | -0.0330432 | 0.0234563 | 0.16 | 16920 | 45.31326528 | 0.000207102 |
| rs11636952 | 15 | 75114322 | C/T | LMAN1L | -0.055 | 0.0094 | 4.21E-09 | 218754 | 0.0253895 | 0.011798 | 0.031 | 16920 | 34.23463494 | 0.000156475 |
| rs11773825 | 7 | 150075576 | A/C | REPIN1\|\|ZNF775 | -0.0627 | 0.0104 | 1.37E-09 | 218754 | 0.0148858 | 0.0122578 | 0.22 | 16920 | 36.34665364 | 0.000166127 |
| rs12567136 | 1 | 11883731 | C/T | CLCN6 | -0.1242 | 0.013 | 1.22E-21 | 218754 | -0.0354539 | 0.0145142 | 0.015 | 16920 | 91.27514182 | 0.00041708 |
| rs1275984 | 2 | 26911509 | A/C | CIB4\|\|KCNK3 | -0.0953 | 0.0092 | 3.38E-25 | 218754 | -0.0202719 | 0.0111013 | 0.068 | 16920 | 107.3015946 | 0.000490277 |
| rs12828438 | 12 | 12883570 | A/G | CDKN1B\|\|MIRN613 | -0.0606 | 0.0092 | 5.01E-11 | 218754 | -0.019252 | 0.0110359 | 0.081 | 16920 | 43.38759954 | 0.000198302 |
| rs13112725 | 4 | 106911742 | C/G | NPNT\|\|MGC16169 | 0.0697 | 0.0121 | 9.34E-09 | 218754 | 0.0121876 | 0.0124733 | 0.33 | 16920 | 33.18110501 | 0.000151661 |
| rs1317181 | 1 | 230873488 | T/G | AGT\|\|CAPN9 | 0.0701 | 0.0112 | 4.04E-10 | 218754 | -0.00454161 | 0.0130711 | 0.73 | 16920 | 39.1738287 | 0.000179047 |
| rs1374264 | 2 | 164999883 | C/A | FIGN\|\|LOC100129745 | -0.0603 | 0.0092 | 6.95E-11 | 218754 | -0.0307804 | 0.010809 | 0.0044 | 16920 | 42.95908266 | 0.000196344 |
| rs145153053 | 17 | 45138033 | A/G | N/A | 0.0707 | 0.0115 | 7.54E-10 | 218754 | 0.0412292 | 0.0143001 | 0.0039 | 16920 | 37.79542004 | 0.000172748 |
| rs149150643 | 4 | 185877562 | C/T | N/A | -0.1328 | 0.0222 | 2.27E-09 | 218754 | -0.0484717 | 0.0248773 | 0.051 | 16920 | 35.78378127 | 0.000163555 |
| rs16853076 | 3 | 168774754 | C/T | C3orf50\|\|EVI1 | -0.1096 | 0.0192 | 1.06E-08 | 218754 | -0.00167397 | 0.0205979 | 0.94 | 16920 | 32.58477153 | 0.000148935 |
| rs1888693 | 10 | 18440444 | G/A | CACNB2 | 0.06 | 0.0094 | 1.45E-10 | 218754 | 0.00296268 | 0.0113018 | 0.79 | 16920 | 40.74204489 | 0.000186213 |
| rs1894400 | 15 | 91428955 | C/T | FES | 0.0834 | 0.0103 | 4.97E-16 | 218754 | -0.0118719 | 0.011591 | 0.31 | 16920 | 65.5622246 | 0.00029962 |
| rs1980235 | 12 | 90110782 | G/A | LOC643153\|\|MRPL2P1 | 0.0663 | 0.0106 | 4.59E-10 | 218754 | 0.00715085 | 0.0115788 | 0.54 | 16920 | 39.12112684 | 0.000178806 |
| rs198833 | 6 | 26114508 | A/G | HIST1H1T\|\|HIST1H2BC | -0.0885 | 0.0146 | 1.19E-09 | 218754 | 0.0141298 | 0.0149716 | 0.35 | 16920 | 36.74319006 | 0.000167939 |
| rs2274224 | 10 | 96039597 | C/G | PLCE1/LOC100128054 | -0.0751 | 0.0096 | 5.64E-15 | 218754 | 0.012548 | 0.0108818 | 0.25 | 16920 | 61.19746566 | 0.000279679 |
| rs2392929 | 7 | 106414069 | G/T | FLJ36031\|\|PIK3CG | 0.062 | 0.01 | 5.21E-10 | 218754 | -0.00129163 | 0.0135393 | 0.92 | 16920 | 38.43964855 | 0.000175692 |
| rs2643826 | 3 | 27562988 | C/T | SLC4A7\|\|LOC643634 | 0.0627 | 0.0093 | 1.75E-11 | 218754 | 0.0107733 | 0.0108659 | 0.32 | 16920 | 45.4532785 | 0.000207741 |
| rs2704368 | 2 | 164443803 | A/G | KCNH7\|\|FIGN | -0.0802 | 0.0124 | 9.52E-11 | 218754 | 0.00122734 | 0.0179057 | 0.95 | 16920 | 41.83130329 | 0.000191191 |
| rs2782981 | 10 | 115781547 | C/T | NHLRC2\|\|LOC100132839 | 0.0799 | 0.0096 | 8.71E-17 | 218754 | 0.00970217 | 0.0120203 | 0.42 | 16920 | 69.27030852 | 0.000316561 |
| rs35427 | 12 | 115556307 | T/G | TBX3\|\|LOC100129020 | -0.0592 | 0.0096 | 5.91E-10 | 218754 | -0.0344645 | 0.0113742 | 0.0024 | 16920 | 38.0274301 | 0.000173808 |
| rs35619711 | 17 | 59482169 | -/C | TBX2 | 0.0614 | 0.0102 | 1.65E-09 | 218754 | 0.0171054 | 0.0121859 | 0.16 | 16920 | 36.23534729 | 0.000165618 |
| rs3790604 | 1 | 113046879 | A/C | WNT2B | 0.1321 | 0.0121 | 1.40E-27 | 218754 | 0.0381559 | 0.0205297 | 0.063 | 16920 | 119.1875586 | 0.000544556 |
| rs3796585 | 4 | 156639174 | G/A | GUCY1A3 | -0.0751 | 0.0096 | 4.48E-15 | 218754 | 0.0211629 | 0.0110957 | 0.056 | 16920 | 61.19746566 | 0.000279679 |
| rs4371736 | 5 | 157877453 | C/G | LOC100130177\|\|EBF1 | -0.0527 | 0.0094 | 2.09E-08 | 218754 | 0.0132329 | 0.0110102 | 0.23 | 16920 | 31.43124274 | 0.000143664 |
| rs4685218 | 3 | 14894140 | C/T | FGD5 | 0.0984 | 0.0153 | 1.30E-10 | 218754 | -0.0165344 | 0.0187763 | 0.38 | 16920 | 41.3621747 | 0.000189047 |
| rs557675 | 11 | 65566719 | T/G | OVOL1\|\|SNX32 | -0.0613 | 0.0093 | 5.42E-11 | 218754 | 0.0189245 | 0.0109004 | 0.083 | 16920 | 43.44612839 | 0.00019857 |
| rs62426324 | 6 | 127142458 | C/T | LOC442257\|\|RSPO3 | 0.0528 | 0.0091 | 7.13E-09 | 218754 | 0.00976898 | 0.0108788 | 0.37 | 16920 | 33.66519154 | 0.000153873 |
| rs62434119 | 6 | 150984431 | C/T | PLEKHG1 | -0.1345 | 0.0172 | 6.23E-15 | 218754 | -0.0289215 | 0.0207126 | 0.16 | 16920 | 61.14820378 | 0.000279454 |
| rs62455829 | 7 | 70045931 | A/G | AUTS2 | -0.0598 | 0.0103 | 5.68E-09 | 218754 | 0.0115118 | 0.0139791 | 0.41 | 16920 | 33.70729857 | 0.000154065 |
| rs629042 | 13 | 22318506 | C/G | FGF9\|\|FTHL7 | 0.0595 | 0.0093 | 1.36E-10 | 218754 | 0.022358 | 0.0109941 | 0.042 | 16920 | 40.93210351 | 0.000187081 |
| rs6668768 | 1 | 25136203 | C/T | CLIC4 | 0.0668 | 0.0111 | 1.87E-09 | 218754 | -0.0118041 | 0.0162721 | 0.47 | 16920 | 36.21620975 | 0.000165531 |
| rs6860901 | 5 | 127871750 | C/T | FBN2 | 0.0682 | 0.01 | 7.68E-12 | 218754 | 0.0115637 | 0.0116611 | 0.32 | 16920 | 46.51197475 | 0.000212579 |
| rs6918791 | 6 | 126218961 | C/G | NCOA7 | 0.0574 | 0.0103 | 2.15E-08 | 218754 | 0.00495843 | 0.0121072 | 0.68 | 16920 | 31.05598904 | 0.000141949 |
| rs7123467 | 11 | 10155174 | C/G | SBF2 | 0.0682 | 0.0114 | 2.47E-09 | 218754 | 0.0129262 | 0.0165083 | 0.43 | 16920 | 35.78945426 | 0.000163581 |
| rs7134677 | 12 | 54441498 | C/T | HOXC4 | -0.0748 | 0.0094 | 2.13E-15 | 218754 | -0.0202924 | 0.0118532 | 0.087 | 16920 | 63.32038079 | 0.000289378 |
| rs7483477 | 11 | 1920255 | T/G | LSP1\|\|TNNT3 | 0.0759 | 0.0109 | 3.12E-12 | 218754 | 0.00944724 | 0.0125425 | 0.45 | 16920 | 48.48714191 | 0.000221604 |
| rs7545442 | 1 | 27260783 | C/T | NUDC | 0.098 | 0.0176 | 2.74E-08 | 218754 | 0.0211137 | 0.0202279 | 0.3 | 16920 | 31.00436529 | 0.000141713 |
| rs78302204 | 20 | 57735448 | A/G | N/A | 0.1406 | 0.0122 | 1.03E-30 | 218754 | 0.0221394 | 0.0175707 | 0.21 | 16920 | 132.8149641 | 0.00060678 |
| rs880315 | 1 | 10796866 | T/C | CASZ1 | 0.1028 | 0.0093 | 1.83E-28 | 218754 | 0.0275747 | 0.0114805 | 0.016 | 16920 | 122.1845691 | 0.000558241 |
| rs9899012 | 17 | 61545486 | A/G | CYB561\|\|LOC342541 | -0.1068 | 0.0166 | 1.41E-10 | 218754 | -0.00922151 | 0.0228167 | 0.69 | 16920 | 41.39256683 | 0.000189186 |

Note: Chr, chromosome; A1 and A2 are effect allele and alternative allele, respectively; SE, stantard error; F, F statistics; LVESV, LV end-systolic volume.

**Table S8** Information on instrumental variables of hypertension and LVM.

| SNP | Chr | Hg19 Position | Allele | Nearby Gene | Hypertension | | | | | LVM | | | | | | F | R^2^ |
| --- | --- | --- | --- | --- | --- | --- | --- | --- | --- | --- | --- | --- | --- | --- | --- | --- | --- |
|  |  |  |  |  | BETA | SE | P | N | | BETA | | SE | | P | N |  |  |
| rs10059884 | 32832474 | 5 | A/C | C5orf23\|\|LOC340113 | 0.0651 | 0.0092 | 1.92E-12 | 218754 | 0.0161398 | | 0.0109877 | | 0.14 | | 16920 | 50.07054883 | 0.000228839 |
| rs10206833 | 159462894 | 2 | A/G | PKP4 | -0.079 | 0.0134 | 3.56E-09 | 218754 | -0.00517144 | | 0.018948 | | 0.78 | | 16920 | 34.75686645 | 0.000158862 |
| rs10217559 | 112751571 | 9 | C/T | PALM2-AKAP2 | 0.0584 | 0.0099 | 3.87E-09 | 218754 | 0.0043552 | | 0.0116284 | | 0.71 | | 16920 | 34.79776368 | 0.000159049 |
| rs11065837 | 111684253 | 12 | A/G | CUX2 | -0.0721 | 0.0108 | 2.49E-11 | 218754 | 0.0141266 | | 0.0128353 | | 0.27 | | 16920 | 44.5675795 | 0.000203694 |
| rs11564041 | 27324368 | 7 | C/T | LOC392008 | -0.1003 | 0.0149 | 1.52E-11 | 218754 | -0.043908 | | 0.0234981 | | 0.062 | | 16920 | 45.31326528 | 0.000207102 |
| rs11636952 | 75114322 | 15 | C/T | LMAN1L | -0.055 | 0.0094 | 4.21E-09 | 218754 | -0.00249965 | | 0.0118116 | | 0.83 | | 16920 | 34.23463494 | 0.000156475 |
| rs11773825 | 150075576 | 7 | A/C | REPIN1\|\|ZNF775 | -0.0627 | 0.0104 | 1.37E-09 | 218754 | 0.00718603 | | 0.0122823 | | 0.56 | | 16920 | 36.34665364 | 0.000166127 |
| rs1275984 | 26911509 | 2 | A/C | CIB4\|\|KCNK3 | -0.0953 | 0.0092 | 3.38E-25 | 218754 | -0.0144696 | | 0.011127 | | 0.19 | | 16920 | 107.3015946 | 0.000490277 |
| rs12828438 | 12883570 | 12 | A/G | CDKN1B\|\|MIRN613 | -0.0606 | 0.0092 | 5.01E-11 | 218754 | -0.019943 | | 0.0110494 | | 0.071 | | 16920 | 43.38759954 | 0.000198302 |
| rs13112725 | 106911742 | 4 | C/G | NPNT\|\|MGC16169 | 0.0697 | 0.0121 | 9.34E-09 | 218754 | 0.0194174 | | 0.0125047 | | 0.12 | | 16920 | 33.18110501 | 0.000151661 |
| rs1317181 | 230873488 | 1 | T/G | AGT\|\|CAPN9 | 0.0701 | 0.0112 | 4.04E-10 | 218754 | 0.0264836 | | 0.0130987 | | 0.043 | | 16920 | 39.1738287 | 0.000179047 |
| rs145153053 | 45138033 | 17 | A/G | N/A | 0.0707 | 0.0115 | 7.54E-10 | 218754 | 0.0242628 | | 0.0143141 | | 0.09 | | 16920 | 37.79542004 | 0.000172748 |
| rs149150643 | 185877562 | 4 | C/T | N/A | -0.1328 | 0.0222 | 2.27E-09 | 218754 | -0.0504254 | | 0.0249398 | | 0.043 | | 16920 | 35.78378127 | 0.000163555 |
| rs16853076 | 168774754 | 3 | C/T | C3orf50\|\|EVI1 | -0.1096 | 0.0192 | 1.06E-08 | 218754 | -0.0175261 | | 0.0206364 | | 0.4 | | 16920 | 32.58477153 | 0.000148935 |
| rs1888693 | 18440444 | 10 | G/A | CACNB2 | 0.06 | 0.0094 | 1.45E-10 | 218754 | 0.00574728 | | 0.0113257 | | 0.61 | | 16920 | 40.74204489 | 0.000186213 |
| rs1894400 | 91428955 | 15 | C/T | FES | 0.0834 | 0.0103 | 4.97E-16 | 218754 | 0.0214446 | | 0.0116036 | | 0.065 | | 16920 | 65.5622246 | 0.00029962 |
| rs1980235 | 90110782 | 12 | G/A | LOC643153\|\|MRPL2P1 | 0.0663 | 0.0106 | 4.59E-10 | 218754 | 0.030421 | | 0.0115945 | | 0.0087 | | 16920 | 39.12112684 | 0.000178806 |
| rs198833 | 26114508 | 6 | A/G | HIST1H1T\|\|HIST1H2BC | -0.0885 | 0.0146 | 1.19E-09 | 218754 | 0.00265105 | | 0.0150027 | | 0.86 | | 16920 | 36.74319006 | 0.000167939 |
| rs2274224 | 96039597 | 10 | C/G | PLCE1/LOC100128054 | -0.0751 | 0.0096 | 5.64E-15 | 218754 | 0.0146478 | | 0.0109043 | | 0.18 | | 16920 | 61.19746566 | 0.000279679 |
| rs2392929 | 106414069 | 7 | G/T | FLJ36031\|\|PIK3CG | 0.062 | 0.01 | 5.21E-10 | 218754 | 0.00975254 | | 0.0135667 | | 0.47 | | 16920 | 38.43964855 | 0.000175692 |
| rs2643826 | 27562988 | 3 | C/T | SLC4A7\|\|LOC643634 | 0.0627 | 0.0093 | 1.75E-11 | 218754 | 0.0225925 | | 0.0108864 | | 0.038 | | 16920 | 45.4532785 | 0.000207741 |
| rs2704368 | 164443803 | 2 | A/G | KCNH7\|\|FIGN | -0.0802 | 0.0124 | 9.52E-11 | 218754 | -0.0115261 | | 0.0179488 | | 0.52 | | 16920 | 41.83130329 | 0.000191191 |
| rs2782981 | 115781547 | 10 | C/T | NHLRC2\|\|LOC100132839 | 0.0799 | 0.0096 | 8.71E-17 | 218754 | -0.00196882 | | 0.012046 | | 0.87 | | 16920 | 69.27030852 | 0.000316561 |
| rs35619711 | 59482169 | 17 | -/C | TBX2 | 0.0614 | 0.0102 | 1.65E-09 | 218754 | 0.0138148 | | 0.0121967 | | 0.26 | | 16920 | 36.23534729 | 0.000165618 |
| rs3790604 | 113046879 | 1 | A/C | WNT2B | 0.1321 | 0.0121 | 1.40E-27 | 218754 | 0.0440297 | | 0.0205705 | | 0.032 | | 16920 | 119.1875586 | 0.000544556 |
| rs3796585 | 156639174 | 4 | G/A | GUCY1A3 | -0.0751 | 0.0096 | 4.48E-15 | 218754 | 0.0156454 | | 0.0111213 | | 0.16 | | 16920 | 61.19746566 | 0.000279679 |
| rs4371736 | 157877453 | 5 | C/G | LOC100130177\|\|EBF1 | -0.0527 | 0.0094 | 2.09E-08 | 218754 | 0.0234931 | | 0.0110419 | | 0.033 | | 16920 | 31.43124274 | 0.000143664 |
| rs4685218 | 14894140 | 3 | C/T | FGD5 | 0.0984 | 0.0153 | 1.30E-10 | 218754 | 0.00342116 | | 0.0188136 | | 0.86 | | 16920 | 41.3621747 | 0.000189047 |
| rs557675 | 65566719 | 11 | T/G | OVOL1\|\|SNX32 | -0.0613 | 0.0093 | 5.42E-11 | 218754 | -0.00817629 | | 0.0109214 | | 0.45 | | 16920 | 43.44612839 | 0.00019857 |
| rs62426324 | 127142458 | 6 | C/T | LOC442257\|\|RSPO3 | 0.0528 | 0.0091 | 7.13E-09 | 218754 | 0.0282674 | | 0.0109006 | | 0.0095 | | 16920 | 33.66519154 | 0.000153873 |
| rs62434119 | 150984431 | 6 | C/T | PLEKHG1 | -0.1345 | 0.0172 | 6.23E-15 | 218754 | -0.0260407 | | 0.0207588 | | 0.21 | | 16920 | 61.14820378 | 0.000279454 |
| rs62455829 | 70045931 | 7 | A/G | AUTS2 | -0.0598 | 0.0103 | 5.68E-09 | 218754 | 0.016956 | | 0.0140064 | | 0.23 | | 16920 | 33.70729857 | 0.000154065 |
| rs629042 | 22318506 | 13 | C/G | FGF9\|\|FTHL7 | 0.0595 | 0.0093 | 1.36E-10 | 218754 | 0.0210151 | | 0.0110063 | | 0.056 | | 16920 | 40.93210351 | 0.000187081 |
| rs6668768 | 25136203 | 1 | C/T | CLIC4 | 0.0668 | 0.0111 | 1.87E-09 | 218754 | 0.00946196 | | 0.0163058 | | 0.56 | | 16920 | 36.21620975 | 0.000165531 |
| rs6860901 | 127871750 | 5 | C/T | FBN2 | 0.0682 | 0.01 | 7.68E-12 | 218754 | 0.000826215 | | 0.0116953 | | 0.94 | | 16920 | 46.51197475 | 0.000212579 |
| rs6918791 | 126218961 | 6 | C/G | NCOA7 | 0.0574 | 0.0103 | 2.15E-08 | 218754 | 0.0134688 | | 0.0121326 | | 0.27 | | 16920 | 31.05598904 | 0.000141949 |
| rs7123467 | 10155174 | 11 | C/G | SBF2 | 0.0682 | 0.0114 | 2.47E-09 | 218754 | 0.00533873 | | 0.0165384 | | 0.75 | | 16920 | 35.78945426 | 0.000163581 |
| rs7134677 | 54441498 | 12 | C/T | HOXC4 | -0.0748 | 0.0094 | 2.13E-15 | 218754 | -0.0184927 | | 0.0118673 | | 0.12 | | 16920 | 63.32038079 | 0.000289378 |
| rs7483477 | 1920255 | 11 | T/G | LSP1\|\|TNNT3 | 0.0759 | 0.0109 | 3.12E-12 | 218754 | 0.0286388 | | 0.0125654 | | 0.023 | | 16920 | 48.48714191 | 0.000221604 |
| rs7545442 | 27260783 | 1 | C/T | NUDC | 0.098 | 0.0176 | 2.74E-08 | 218754 | 0.0474699 | | 0.0202711 | | 0.019 | | 16920 | 31.00436529 | 0.000141713 |
| rs78302204 | 57735448 | 20 | A/G | N/A | 0.1406 | 0.0122 | 1.03E-30 | 218754 | 0.0397756 | | 0.0175986 | | 0.024 | | 16920 | 132.8149641 | 0.00060678 |
| rs880315 | 10796866 | 1 | T/C | CASZ1 | 0.1028 | 0.0093 | 1.83E-28 | 218754 | 0.0327285 | | 0.0115051 | | 0.0044 | | 16920 | 122.1845691 | 0.000558241 |
| rs9899012 | 61545486 | 17 | A/G | CYB561\|\|LOC342541 | -0.1068 | 0.0166 | 1.41E-10 | 218754 | -0.0302076 | | 0.0228409 | | 0.19 | | 16920 | 41.39256683 | 0.000189186 |

Note: Chr, chromosome;A1 and A2 are effect allele and alternative allele, respectively; SE, stantard error; F, F statistics; LVM, LV mass.

**Table S9** Information on instrumental variables of hypertension and LVMVR.

| SNP | Chr | Hg19 Position | A1/A2 | Nearby Gene | Hypertension | | | | LVMVR | | | | F | R^2^ |
| --- | --- | --- | --- | --- | --- | --- | --- | --- | --- | --- | --- | --- | --- | --- |
|  |  |  |  |  | BETA | SE | P | N | BETA | SE | P | N |  |  |
| rs10059884 | 5 | 32832474 | A/C | C5orf23\|\|LOC340113 | 0.0651 | 0.0092 | 1.92E-12 | 218754 | 0.00895753 | 0.0109981 | 0.42 | 16884 | 50.07054883 | 0.000228839 |
| rs10206833 | 2 | 159462894 | A/G | PKP4 | -0.079 | 0.0134 | 3.56E-09 | 218754 | -0.0166435 | 0.0189745 | 0.38 | 16884 | 34.75686645 | 0.000158862 |
| rs10217559 | 9 | 112751571 | C/T | PALM2-AKAP2 | 0.0584 | 0.0099 | 3.87E-09 | 218754 | -0.00839699 | 0.0116348 | 0.47 | 16884 | 34.79776368 | 0.000159049 |
| rs11065837 | 12 | 111684253 | A/G | CUX2 | -0.0721 | 0.0108 | 2.49E-11 | 218754 | -0.0279162 | 0.0128452 | 0.03 | 16884 | 44.5675795 | 0.000203694 |
| rs11564041 | 7 | 27324368 | C/T | LOC392008 | -0.1003 | 0.0149 | 1.52E-11 | 218754 | -0.00745968 | 0.0235302 | 0.75 | 16884 | 45.31326528 | 0.000207102 |
| rs11636952 | 15 | 75114322 | C/T | LMAN1L | -0.055 | 0.0094 | 4.21E-09 | 218754 | -0.0244416 | 0.0118252 | 0.039 | 16884 | 34.23463494 | 0.000156475 |
| rs11773825 | 7 | 150075576 | A/C | REPIN1\|\|ZNF775 | -0.0627 | 0.0104 | 1.37E-09 | 218754 | -0.0199533 | 0.0122952 | 0.1 | 16884 | 36.34665364 | 0.000166127 |
| rs12567136 | 1 | 11883731 | C/T | CLCN6 | -0.1242 | 0.013 | 1.22E-21 | 218754 | -0.00826515 | 0.0145473 | 0.57 | 16884 | 91.27514182 | 0.00041708 |
| rs1275984 | 2 | 26911509 | A/C | CIB4\|\|KCNK3 | -0.0953 | 0.0092 | 3.38E-25 | 218754 | -0.00714282 | 0.0111501 | 0.52 | 16884 | 107.3015946 | 0.000490277 |
| rs12828438 | 12 | 12883570 | A/G | CDKN1B\|\|MIRN613 | -0.0606 | 0.0092 | 5.01E-11 | 218754 | -0.00481041 | 0.0110538 | 0.66 | 16884 | 43.38759954 | 0.000198302 |
| rs13112725 | 4 | 106911742 | C/G | NPNT\|\|MGC16169 | 0.0697 | 0.0121 | 9.34E-09 | 218754 | 0.0217501 | 0.0125148 | 0.082 | 16884 | 33.18110501 | 0.000151661 |
| rs1317181 | 1 | 230873488 | T/G | AGT\|\|CAPN9 | 0.0701 | 0.0112 | 4.04E-10 | 218754 | 0.00237713 | 0.0131137 | 0.86 | 16884 | 39.1738287 | 0.000179047 |
| rs1374264 | 2 | 164999883 | C/A | FIGN\|\|LOC100129745 | -0.0603 | 0.0092 | 6.95E-11 | 218754 | -0.00945642 | 0.0108536 | 0.38 | 16884 | 42.95908266 | 0.000196344 |
| rs145153053 | 17 | 45138033 | A/G | N/A | 0.0707 | 0.0115 | 7.54E-10 | 218754 | -0.0178015 | 0.0143339 | 0.21 | 16884 | 37.79542004 | 0.000172748 |
| rs149150643 | 4 | 185877562 | C/T | N/A | -0.1328 | 0.0222 | 2.27E-09 | 218754 | -0.000430391 | 0.0249641 | 0.99 | 16884 | 35.78378127 | 0.000163555 |
| rs16853076 | 3 | 168774754 | C/T | C3orf50\|\|EVI1 | -0.1096 | 0.0192 | 1.06E-08 | 218754 | -0.0309435 | 0.020675 | 0.13 | 16884 | 32.58477153 | 0.000148935 |
| rs1888693 | 10 | 18440444 | G/A | CACNB2 | 0.06 | 0.0094 | 1.45E-10 | 218754 | 0.0041372 | 0.0113508 | 0.72 | 16884 | 40.74204489 | 0.000186213 |
| rs1894400 | 15 | 91428955 | C/T | FES | 0.0834 | 0.0103 | 4.97E-16 | 218754 | 0.0222568 | 0.011618 | 0.055 | 16884 | 65.5622246 | 0.00029962 |
| rs1980235 | 12 | 90110782 | G/A | LOC643153\|\|MRPL2P1 | 0.0663 | 0.0106 | 4.59E-10 | 218754 | 0.0250484 | 0.0116048 | 0.031 | 16884 | 39.12112684 | 0.000178806 |
| rs198833 | 6 | 26114508 | A/G | HIST1H1T\|\|HIST1H2BC | -0.0885 | 0.0146 | 1.19E-09 | 218754 | -0.0171344 | 0.0149926 | 0.25 | 16884 | 36.74319006 | 0.000167939 |
| rs2274224 | 10 | 96039597 | C/G | PLCE1/LOC100128054 | -0.0751 | 0.0096 | 5.64E-15 | 218754 | -0.0095641 | 0.0109207 | 0.38 | 16884 | 61.19746566 | 0.000279679 |
| rs2392929 | 7 | 106414069 | G/T | FLJ36031\|\|PIK3CG | 0.062 | 0.01 | 5.21E-10 | 218754 | -0.0168727 | 0.013588 | 0.21 | 16884 | 38.43964855 | 0.000175692 |
| rs2643826 | 3 | 27562988 | C/T | SLC4A7\|\|LOC643634 | 0.0627 | 0.0093 | 1.75E-11 | 218754 | 0.0131648 | 0.0109058 | 0.23 | 16884 | 45.4532785 | 0.000207741 |
| rs2704368 | 2 | 164443803 | A/G | KCNH7\|\|FIGN | -0.0802 | 0.0124 | 9.52E-11 | 218754 | -0.0234681 | 0.0179854 | 0.19 | 16884 | 41.83130329 | 0.000191191 |
| rs2782981 | 10 | 115781547 | C/T | NHLRC2\|\|LOC100132839 | 0.0799 | 0.0096 | 8.71E-17 | 218754 | 0.00635069 | 0.012068 | 0.6 | 16884 | 69.27030852 | 0.000316561 |
| rs35427 | 12 | 115556307 | T/G | TBX3\|\|LOC100129020 | -0.0592 | 0.0096 | 5.91E-10 | 218754 | 0.0197925 | 0.0113957 | 0.082 | 16884 | 38.0274301 | 0.000173808 |
| rs35619711 | 17 | 59482169 | -/C | TBX2 | 0.0614 | 0.0102 | 1.65E-09 | 218754 | -0.00457138 | 0.0122142 | 0.71 | 16884 | 36.23534729 | 0.000165618 |
| rs3790604 | 1 | 113046879 | A/C | WNT2B | 0.1321 | 0.0121 | 1.40E-27 | 218754 | 0.0100421 | 0.0205941 | 0.63 | 16884 | 119.1875586 | 0.000544556 |
| rs3796585 | 4 | 156639174 | G/A | GUCY1A3 | -0.0751 | 0.0096 | 4.48E-15 | 218754 | -0.00669959 | 0.0111228 | 0.55 | 16884 | 61.19746566 | 0.000279679 |
| rs4371736 | 5 | 157877453 | C/G | LOC100130177\|\|EBF1 | -0.0527 | 0.0094 | 2.09E-08 | 218754 | 0.00824831 | 0.0110468 | 0.46 | 16884 | 31.43124274 | 0.000143664 |
| rs4685218 | 3 | 14894140 | C/T | FGD5 | 0.0984 | 0.0153 | 1.30E-10 | 218754 | 0.0339828 | 0.0188527 | 0.071 | 16884 | 41.3621747 | 0.000189047 |
| rs557675 | 11 | 65566719 | T/G | OVOL1\|\|SNX32 | -0.0613 | 0.0093 | 5.42E-11 | 218754 | -0.0356357 | 0.010933 | 0.0011 | 16884 | 43.44612839 | 0.00019857 |
| rs62426324 | 6 | 127142458 | C/T | LOC442257\|\|RSPO3 | 0.0528 | 0.0091 | 7.13E-09 | 218754 | 0.02872 | 0.0108949 | 0.0084 | 16884 | 33.66519154 | 0.000153873 |
| rs62434119 | 6 | 150984431 | C/T | PLEKHG1 | -0.1345 | 0.0172 | 6.23E-15 | 218754 | 0.0127355 | 0.0207349 | 0.54 | 16884 | 61.14820378 | 0.000279454 |
| rs62455829 | 7 | 70045931 | A/G | AUTS2 | -0.0598 | 0.0103 | 5.68E-09 | 218754 | -0.018081 | 0.0140239 | 0.2 | 16884 | 33.70729857 | 0.000154065 |
| rs629042 | 13 | 22318506 | C/G | FGF9\|\|FTHL7 | 0.0595 | 0.0093 | 1.36E-10 | 218754 | -0.00698109 | 0.0110282 | 0.53 | 16884 | 40.93210351 | 0.000187081 |
| rs6668768 | 1 | 25136203 | C/T | CLIC4 | 0.0668 | 0.0111 | 1.87E-09 | 218754 | 0.0187922 | 0.0163102 | 0.25 | 16884 | 36.21620975 | 0.000165531 |
| rs6860901 | 5 | 127871750 | C/T | FBN2 | 0.0682 | 0.01 | 7.68E-12 | 218754 | -0.00696745 | 0.0117082 | 0.55 | 16884 | 46.51197475 | 0.000212579 |
| rs6918791 | 6 | 126218961 | C/G | NCOA7 | 0.0574 | 0.0103 | 2.15E-08 | 218754 | 0.00771392 | 0.0121228 | 0.52 | 16884 | 31.05598904 | 0.000141949 |
| rs7123467 | 11 | 10155174 | C/G | SBF2 | 0.0682 | 0.0114 | 2.47E-09 | 218754 | -0.0171031 | 0.0165528 | 0.3 | 16884 | 35.78945426 | 0.000163581 |
| rs7134677 | 12 | 54441498 | C/T | HOXC4 | -0.0748 | 0.0094 | 2.13E-15 | 218754 | 0.0110372 | 0.0118761 | 0.35 | 16884 | 63.32038079 | 0.000289378 |
| rs7483477 | 11 | 1920255 | T/G | LSP1\|\|TNNT3 | 0.0759 | 0.0109 | 3.12E-12 | 218754 | -0.00247912 | 0.0125799 | 0.84 | 16884 | 48.48714191 | 0.000221604 |
| rs7545442 | 1 | 27260783 | C/T | NUDC | 0.098 | 0.0176 | 2.74E-08 | 218754 | 0.0101895 | 0.0202786 | 0.62 | 16884 | 31.00436529 | 0.000141713 |
| rs78302204 | 20 | 57735448 | A/G | N/A | 0.1406 | 0.0122 | 1.03E-30 | 218754 | 0.0233445 | 0.0176355 | 0.19 | 16884 | 132.8149641 | 0.00060678 |
| rs880315 | 1 | 10796866 | T/C | CASZ1 | 0.1028 | 0.0093 | 1.83E-28 | 218754 | -0.00610262 | 0.0115141 | 0.6 | 16884 | 122.1845691 | 0.000558241 |
| rs9899012 | 17 | 61545486 | A/G | CYB561\|\|LOC342541 | -0.1068 | 0.0166 | 1.41E-10 | 218754 | 0.0019672 | 0.0228652 | 0.93 | 16884 | 41.39256683 | 0.000189186 |

Note: Chr, chromosome; A1 and A2 are effect allele and alternative allele, respectively; SE, stantard error; F, F statistics; LVMVR, LV mass to end-diastolic volume ratio.

**Table S10** Information on instrumental variables of hypertension and LVEF.

| SNP | Chr | Hg19 Position | A1/A2 | Nearby Gene | Hypertension | | | | LVEF | | | | F | R^2^ |
| --- | --- | --- | --- | --- | --- | --- | --- | --- | --- | --- | --- | --- | --- | --- |
|  |  |  |  |  | BETA | SE | P | N | BETA | SE | P | N |  |  |
| rs10059884 | 5 | 32832474 | A/C | C5orf23\|\|LOC340113 | 0.0651 | 0.0092 | 1.92E-12 | 218754 | -0.0143079 | 0.0110216 | 0.19 | 16923 | 50.07054883 | 0.000228839 |
| rs10206833 | 2 | 159462894 | A/G | PKP4 | -0.079 | 0.0134 | 3.56E-09 | 218754 | 0.000413411 | 0.0190318 | 0.98 | 16923 | 34.75686645 | 0.000158862 |
| rs10217559 | 9 | 112751571 | C/T | PALM2-AKAP2 | 0.0584 | 0.0099 | 3.87E-09 | 218754 | -0.00545814 | 0.0116695 | 0.64 | 16923 | 34.79776368 | 0.000159049 |
| rs11065837 | 12 | 111684253 | A/G | CUX2 | -0.0721 | 0.0108 | 2.49E-11 | 218754 | -0.0276597 | 0.0128893 | 0.032 | 16923 | 44.5675795 | 0.000203694 |
| rs11564041 | 7 | 27324368 | C/T | LOC392008 | -0.1003 | 0.0149 | 1.52E-11 | 218754 | 0.0182392 | 0.0235958 | 0.44 | 16923 | 45.31326528 | 0.000207102 |
| rs11636952 | 15 | 75114322 | C/T | LMAN1L | -0.055 | 0.0094 | 4.21E-09 | 218754 | -0.0230366 | 0.0118602 | 0.052 | 16923 | 34.23463494 | 0.000156475 |
| rs11773825 | 7 | 150075576 | A/C | REPIN1\|\|ZNF775 | -0.0627 | 0.0104 | 1.37E-09 | 218754 | 0.00882266 | 0.0123309 | 0.47 | 16923 | 36.34665364 | 0.000166127 |
| rs12567136 | 1 | 11883731 | C/T | CLCN6 | -0.1242 | 0.013 | 1.22E-21 | 218754 | 0.0102999 | 0.0146047 | 0.48 | 16923 | 91.27514182 | 0.00041708 |
| rs1275984 | 2 | 26911509 | A/C | CIB4\|\|KCNK3 | -0.0953 | 0.0092 | 3.38E-25 | 218754 | 0.0148766 | 0.0111785 | 0.18 | 16923 | 107.3015946 | 0.000490277 |
| rs12828438 | 12 | 12883570 | A/G | CDKN1B\|\|MIRN613 | -0.0606 | 0.0092 | 5.01E-11 | 218754 | 0.0156548 | 0.0110965 | 0.16 | 16923 | 43.38759954 | 0.000198302 |
| rs13112725 | 4 | 106911742 | C/G | NPNT\|\|MGC16169 | 0.0697 | 0.0121 | 9.34E-09 | 218754 | -0.00774938 | 0.0125493 | 0.54 | 16923 | 33.18110501 | 0.000151661 |
| rs1317181 | 1 | 230873488 | T/G | AGT\|\|CAPN9 | 0.0701 | 0.0112 | 4.04E-10 | 218754 | 0.0282764 | 0.0131564 | 0.032 | 16923 | 39.1738287 | 0.000179047 |
| rs1374264 | 2 | 164999883 | C/A | FIGN\|\|LOC100129745 | -0.0603 | 0.0092 | 6.95E-11 | 218754 | 0.0204561 | 0.0108827 | 0.06 | 16923 | 42.95908266 | 0.000196344 |
| rs145153053 | 17 | 45138033 | A/G | N/A | 0.0707 | 0.0115 | 7.54E-10 | 218754 | -0.0204109 | 0.0143698 | 0.16 | 16923 | 37.79542004 | 0.000172748 |
| rs149150643 | 4 | 185877562 | C/T | N/A | -0.1328 | 0.0222 | 2.27E-09 | 218754 | 0.0140637 | 0.0250301 | 0.57 | 16923 | 35.78378127 | 0.000163555 |
| rs16853076 | 3 | 168774754 | C/T | C3orf50\|\|EVI1 | -0.1096 | 0.0192 | 1.06E-08 | 218754 | 0.0190652 | 0.0207377 | 0.36 | 16923 | 32.58477153 | 0.000148935 |
| rs1888693 | 10 | 18440444 | G/A | CACNB2 | 0.06 | 0.0094 | 1.45E-10 | 218754 | -0.00962531 | 0.0113664 | 0.4 | 16923 | 40.74204489 | 0.000186213 |
| rs1894400 | 15 | 91428955 | C/T | FES | 0.0834 | 0.0103 | 4.97E-16 | 218754 | 0.00686005 | 0.0116511 | 0.56 | 16923 | 65.5622246 | 0.00029962 |
| rs1980235 | 12 | 90110782 | G/A | LOC643153\|\|MRPL2P1 | 0.0663 | 0.0106 | 4.59E-10 | 218754 | -0.00683258 | 0.0116423 | 0.56 | 16923 | 39.12112684 | 0.000178806 |
| rs198833 | 6 | 26114508 | A/G | HIST1H1T\|\|HIST1H2BC | -0.0885 | 0.0146 | 1.19E-09 | 218754 | -0.0152832 | 0.0150479 | 0.31 | 16923 | 36.74319006 | 0.000167939 |
| rs2274224 | 10 | 96039597 | C/G | PLCE1/LOC100128054 | -0.0751 | 0.0096 | 5.64E-15 | 218754 | 0.00802997 | 0.0109431 | 0.46 | 16923 | 61.19746566 | 0.000279679 |
| rs2392929 | 7 | 106414069 | G/T | FLJ36031\|\|PIK3CG | 0.062 | 0.01 | 5.21E-10 | 218754 | 0.0292227 | 0.0136221 | 0.032 | 16923 | 38.43964855 | 0.000175692 |
| rs2643826 | 3 | 27562988 | C/T | SLC4A7\|\|LOC643634 | 0.0627 | 0.0093 | 1.75E-11 | 218754 | -0.00708586 | 0.0109385 | 0.52 | 16923 | 45.4532785 | 0.000207741 |
| rs2704368 | 2 | 164443803 | A/G | KCNH7\|\|FIGN | -0.0802 | 0.0124 | 9.52E-11 | 218754 | 0.00248344 | 0.0180306 | 0.89 | 16923 | 41.83130329 | 0.000191191 |
| rs2782981 | 10 | 115781547 | C/T | NHLRC2\|\|LOC100132839 | 0.0799 | 0.0096 | 8.71E-17 | 218754 | -0.0247821 | 0.0120888 | 0.04 | 16923 | 69.27030852 | 0.000316561 |
| rs35427 | 12 | 115556307 | T/G | TBX3\|\|LOC100129020 | -0.0592 | 0.0096 | 5.91E-10 | 218754 | -0.0032052 | 0.011436 | 0.78 | 16923 | 38.0274301 | 0.000173808 |
| rs35619711 | 17 | 59482169 | -/C | TBX2 | 0.0614 | 0.0102 | 1.65E-09 | 218754 | -0.00274041 | 0.0122438 | 0.82 | 16923 | 36.23534729 | 0.000165618 |
| rs3790604 | 1 | 113046879 | A/C | WNT2B | 0.1321 | 0.0121 | 1.40E-27 | 218754 | -0.0260731 | 0.0206618 | 0.21 | 16923 | 119.1875586 | 0.000544556 |
| rs3796585 | 4 | 156639174 | G/A | GUCY1A3 | -0.0751 | 0.0096 | 4.48E-15 | 218754 | -0.0158041 | 0.0111606 | 0.16 | 16923 | 61.19746566 | 0.000279679 |
| rs4371736 | 5 | 157877453 | C/G | LOC100130177\|\|EBF1 | -0.0527 | 0.0094 | 2.09E-08 | 218754 | -0.00386647 | 0.0110769 | 0.73 | 16923 | 31.43124274 | 0.000143664 |
| rs4685218 | 3 | 14894140 | C/T | FGD5 | 0.0984 | 0.0153 | 1.30E-10 | 218754 | -0.0129765 | 0.0189037 | 0.49 | 16923 | 41.3621747 | 0.000189047 |
| rs557675 | 11 | 65566719 | T/G | OVOL1\|\|SNX32 | -0.0613 | 0.0093 | 5.42E-11 | 218754 | -0.0125972 | 0.0109609 | 0.25 | 16923 | 43.44612839 | 0.00019857 |
| rs62426324 | 6 | 127142458 | C/T | LOC442257\|\|RSPO3 | 0.0528 | 0.0091 | 7.13E-09 | 218754 | -0.0183464 | 0.0109337 | 0.093 | 16923 | 33.66519154 | 0.000153873 |
| rs62434119 | 6 | 150984431 | C/T | PLEKHG1 | -0.1345 | 0.0172 | 6.23E-15 | 218754 | 0.0107847 | 0.0208186 | 0.6 | 16923 | 61.14820378 | 0.000279454 |
| rs62455829 | 7 | 70045931 | A/G | AUTS2 | -0.0598 | 0.0103 | 5.68E-09 | 218754 | 0.0118001 | 0.0140642 | 0.4 | 16923 | 33.70729857 | 0.000154065 |
| rs629042 | 13 | 22318506 | C/G | FGF9\|\|FTHL7 | 0.0595 | 0.0093 | 1.36E-10 | 218754 | -0.00777159 | 0.0110524 | 0.48 | 16923 | 40.93210351 | 0.000187081 |
| rs6668768 | 1 | 25136203 | C/T | CLIC4 | 0.0668 | 0.0111 | 1.87E-09 | 218754 | 0.00810557 | 0.0163779 | 0.62 | 16923 | 36.21620975 | 0.000165531 |
| rs6860901 | 5 | 127871750 | C/T | FBN2 | 0.0682 | 0.01 | 7.68E-12 | 218754 | -0.00862948 | 0.0117329 | 0.46 | 16923 | 46.51197475 | 0.000212579 |
| rs6918791 | 6 | 126218961 | C/G | NCOA7 | 0.0574 | 0.0103 | 2.15E-08 | 218754 | -0.00552858 | 0.0121687 | 0.65 | 16923 | 31.05598904 | 0.000141949 |
| rs7123467 | 11 | 10155174 | C/G | SBF2 | 0.0682 | 0.0114 | 2.47E-09 | 218754 | 0.0113582 | 0.0165996 | 0.49 | 16923 | 35.78945426 | 0.000163581 |
| rs7134677 | 12 | 54441498 | C/T | HOXC4 | -0.0748 | 0.0094 | 2.13E-15 | 218754 | 0.00570036 | 0.0119172 | 0.63 | 16923 | 63.32038079 | 0.000289378 |
| rs7483477 | 11 | 1920255 | T/G | LSP1\|\|TNNT3 | 0.0759 | 0.0109 | 3.12E-12 | 218754 | 0.0097268 | 0.0126114 | 0.44 | 16923 | 48.48714191 | 0.000221604 |
| rs7545442 | 1 | 27260783 | C/T | NUDC | 0.098 | 0.0176 | 2.74E-08 | 218754 | 0.0113617 | 0.0203581 | 0.58 | 16923 | 31.00436529 | 0.000141713 |
| rs78302204 | 20 | 57735448 | A/G | N/A | 0.1406 | 0.0122 | 1.03E-30 | 218754 | -0.0121888 | 0.0176664 | 0.49 | 16923 | 132.8149641 | 0.00060678 |
| rs880315 | 1 | 10796866 | T/C | CASZ1 | 0.1028 | 0.0093 | 1.83E-28 | 218754 | 0.000225434 | 0.011556 | 0.98 | 16923 | 122.1845691 | 0.000558241 |
| rs9899012 | 17 | 61545486 | A/G | CYB561\|\|LOC342541 | -0.1068 | 0.0166 | 1.41E-10 | 218754 | -0.0036873 | 0.0229258 | 0.87 | 16923 | 41.39256683 | 0.000189186 |

Note: Chr, chromosome; A1 and A2 are effect allele and alternative allele, respectively; SE, stantard error; F, F statistics; LVEF, LV ejection fraction.


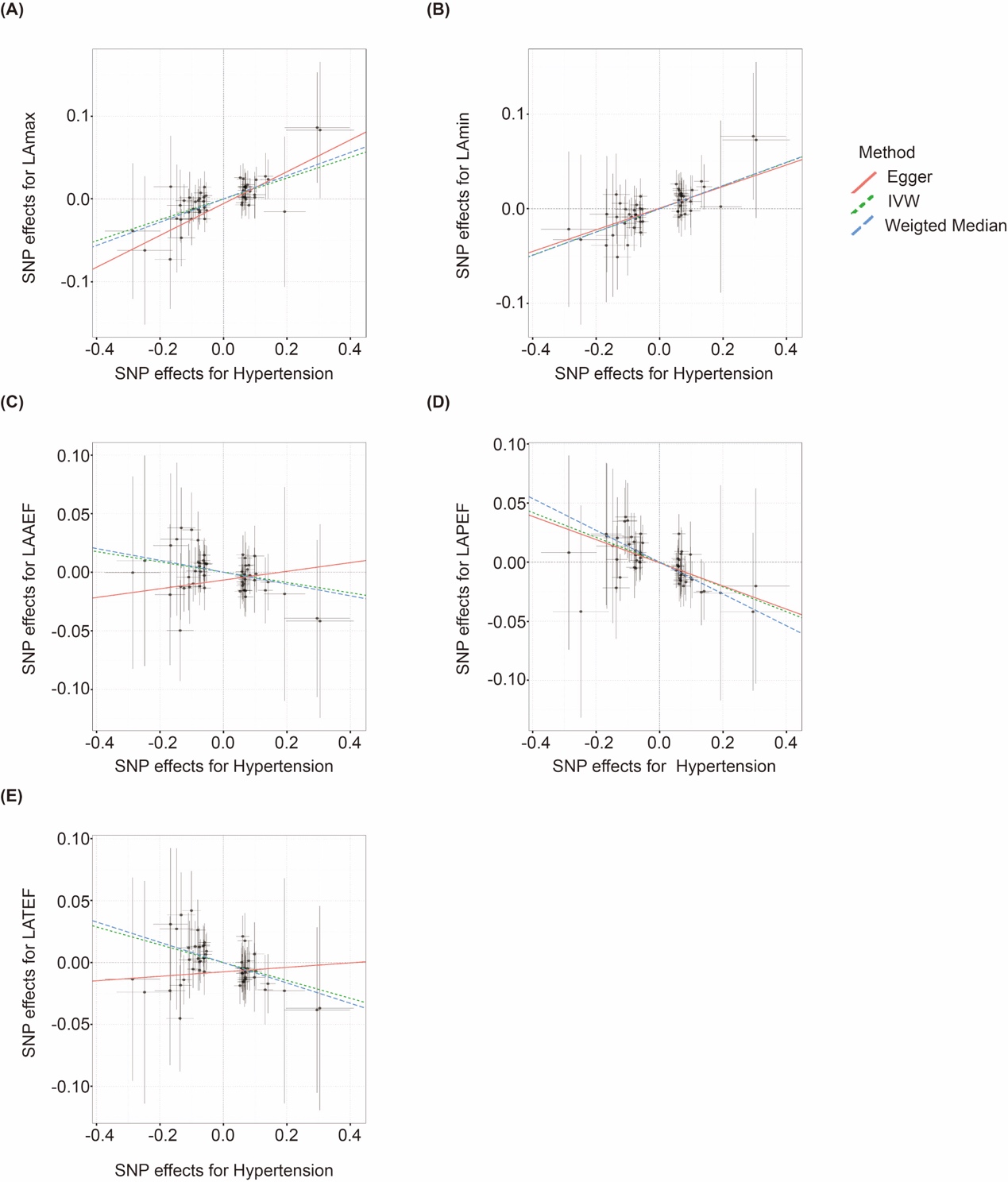


Supplementary Figure 1 Scatter plot of left atrial index.


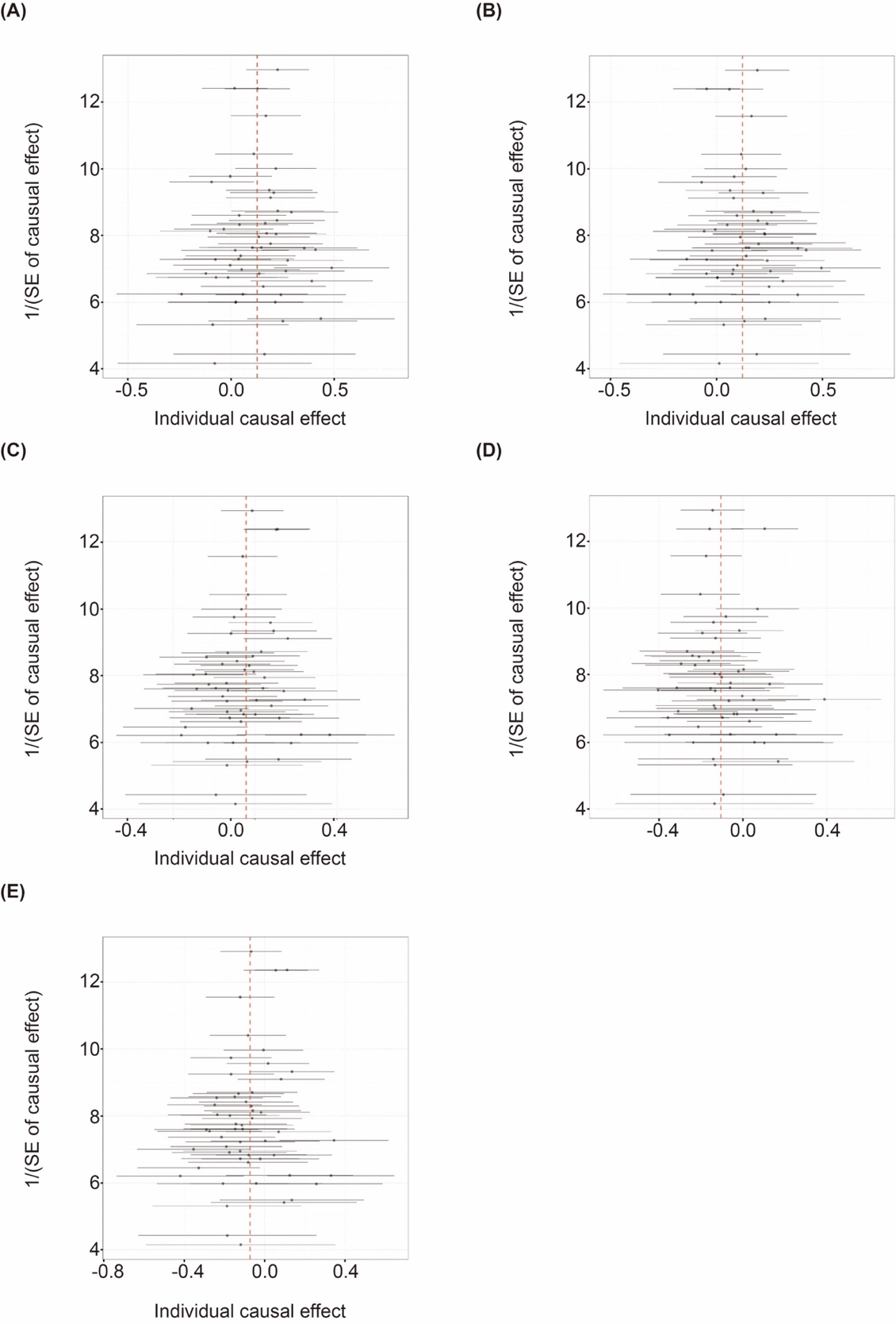


Supplementary Figure 2 Funnel chart of LA index, A is for LAmax; B is for LAmin; C is for LAAEF; D is for LAPEF; E is for LATEF.

LA, left atrial; LAmax, LA maximum volumes; LAmin, LA minimum volumes; LAAEF, LA active emptying fraction; LAPEF, LA passive emptying fraction; LATEF, LA total emptying fraction.


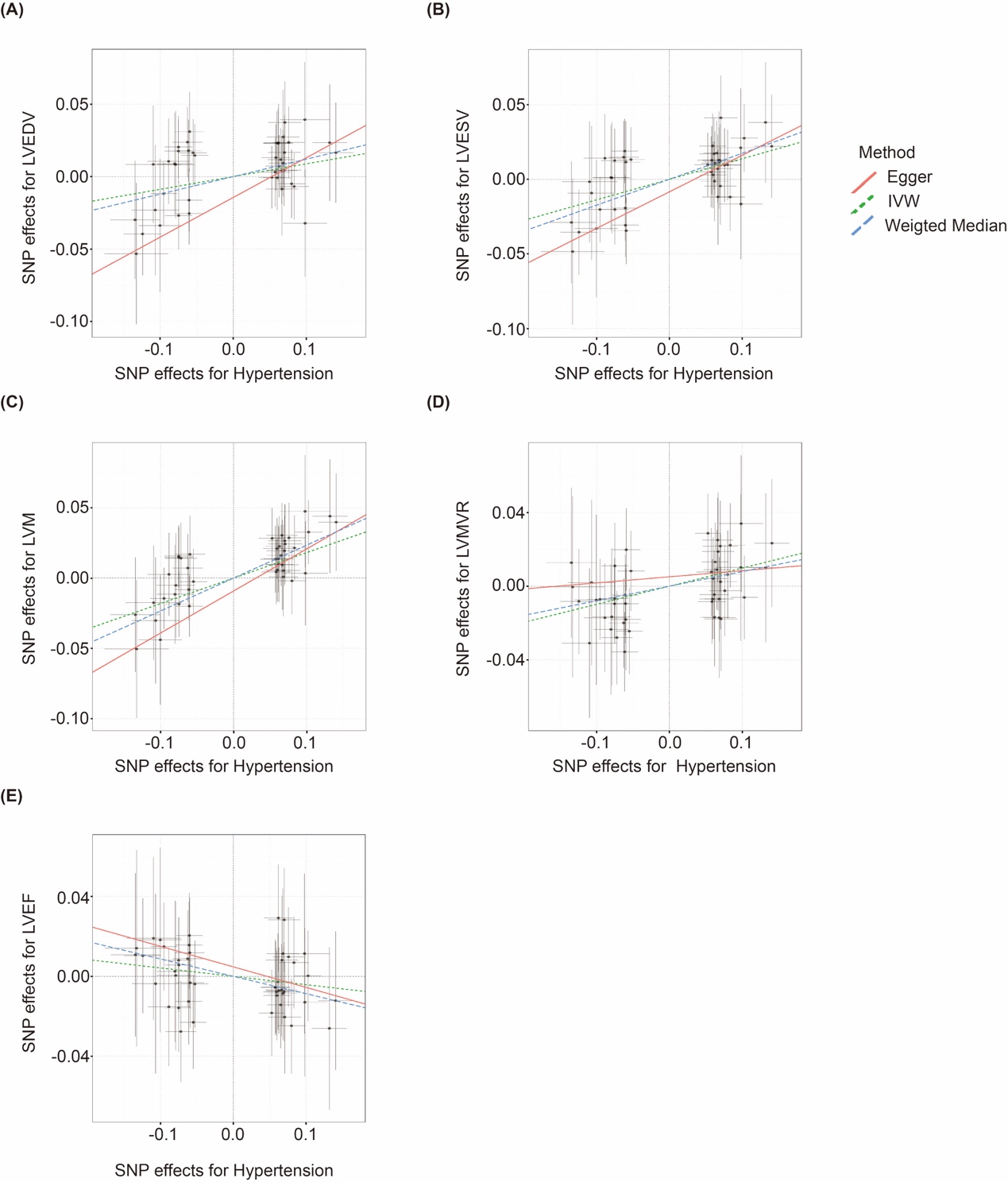


Supplementary Figure 3 Scatter plot of left ventricular index.


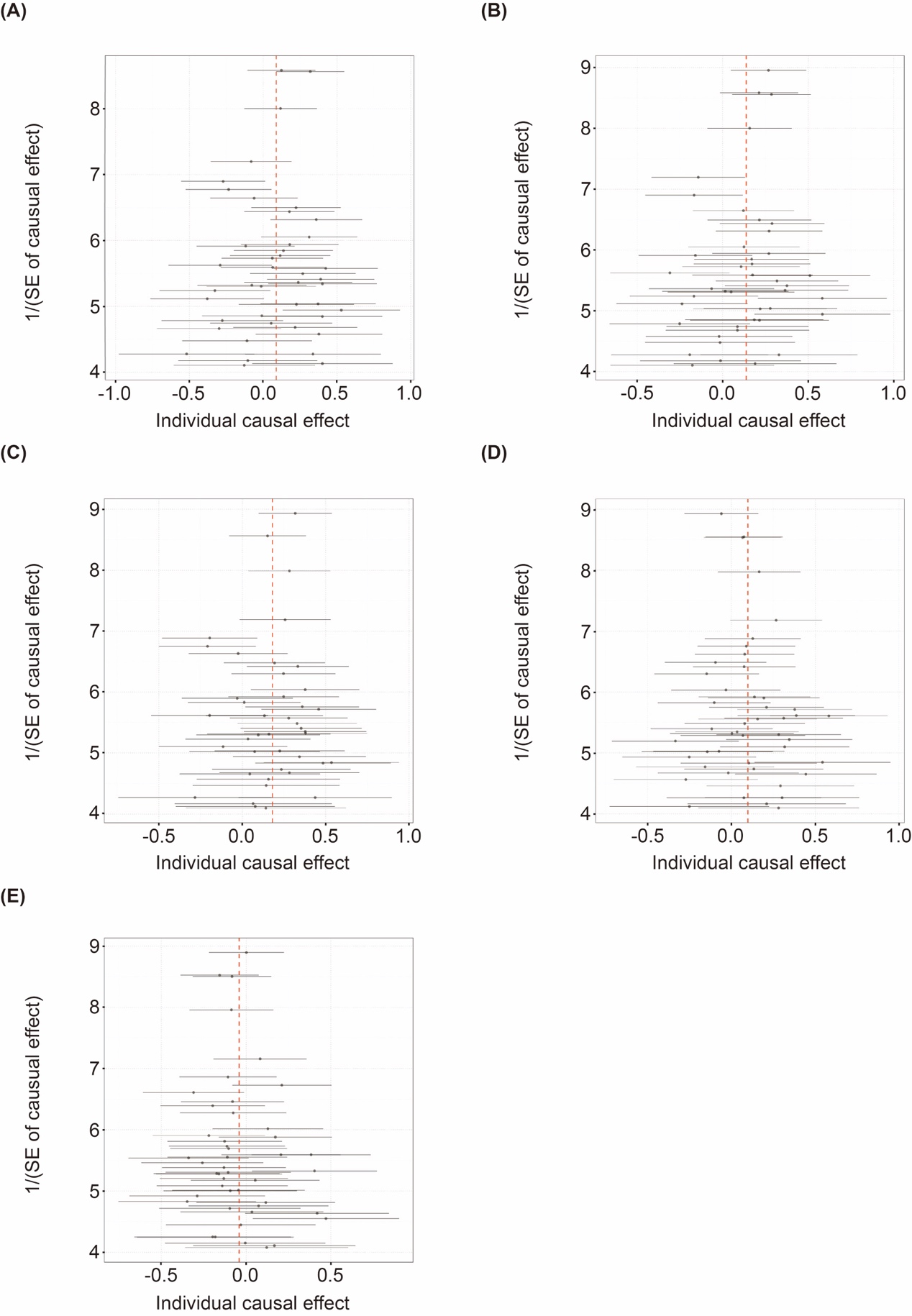


Supplementary Figure 4 Funnel chart of LV index, A is for LVEDV; B is for LVESV; C is for LVM; D is for LVMVR; E is for LVEF.

LV, left ventricular; LVEDV, LV end-diastolic volume; LVESV, LV end-systolic volume, LVM, LV mass, LVMVR, LV mass to end-diastolic volume ratio; LVEF, LV ejection fraction.
